# Tunable Surface Chemistry of Molten Salt Derived MXenes for Data‐Driven Electrochemical Materials Discovery

**DOI:** 10.1002/smll.74471

**Published:** 2026-07-11

**Authors:** Yingrui Wang, Simin Li, Tao Yang, Xinmei Hou, Bo Shen

**Affiliations:** ^1^ Department of Materials Science and Engineering City University of Hong Kong Hong Kong SAR China; ^2^ Institute for Carbon Neutrality Beijing Advanced Innovation Center for Materials Genome Engineering University of Science and Technology Beijing Beijing China

**Keywords:** data‐driven discovery, Lewis acid molten salts, MXene, surface chemistry

## Abstract

The surface chemistry of MXenes is a central factor governing electrochemical performance and has become increasingly complex with the emergence of molten salt etching routes. Compared with conventional fluoride‐derived systems, molten salt derived MXenes exhibit chemically diverse and highly tunable surface states arising from termination chemistry, retained metal species, interfacial evolution, and post‐synthetic regulation. Such synthesis‐dependent complexity creates a strongly coupled design space that motivates data‐driven discovery. In this review, we summarize recent progress in molten salt derived MXenes with emphasis on tunable surface chemistry and its implications for data‐driven design. Surface termination engineering, metal incorporation, heterostructure formation, and post‐synthetic modification are discussed as interconnected surface‐regulation pathways. We further examine how synthesis history, surface chemistry, and electronic structure can be translated into physically meaningful descriptors for machine learning and high‐throughput modeling. Overall, this review establishes a chemistry‐informed framework linking molten salt synthesis, tunable surface chemistry, and data‐driven discovery toward electrochemical applications.

## Graphical abstract

The surface chemistries of MXenes have been largely broadened by Lewis acid melts through expanded compositional diversity and functionalization routes. Such complexity makes data‐driven approaches particularly suitable for accelerated functionality discovery. This review examines surface chemistry from synthetic history to structure‐activity relations, while also summarizes advances in machine learning‐assisted MXene discovery.

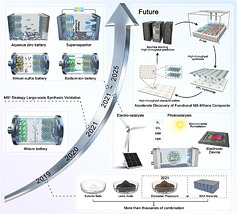

## 1 Introduction

Among the rapidly expanding family of two‐dimensional materials, MXenes have emerged as a versatile class with broad applications in energy storage, catalysis, water purification, and sensing [1]. Their mechanical robustness, metallic conductivity, and layered structure with high and tunable surface area underpin their functional performance across these applications [2]. Importantly, the properties of MXenes are strongly governed by their synthetic process, particularly the resulting surface terminations and interfacial chemistry [3]. As a result, surface chemistry has become a central factor in understanding and designing MXene functionality [4].

MXenes are typically synthesized via selective etching of MAX phase precursors, most commonly through fluoride‐based routes using hydrofluoric acid or in situ generated HF [5]. Beyond removing the A layer, these processes introduce mixed surface terminations (e.g., –O, –OH, and –F), leading to significant variation in the local electronic states of surface metal sites [6]. Together with subsequent functionalization strategies, such surface chemistries govern ion adsorption, charge transfer, and the interfacial environment in energy storage and conversion systems [7]. Furthermore, the diversity of MXene compositions combined with in‐plane or out‐of‐plane ordering and various surface terminations, creates a vast design space [8].

**FIGURE 1:**
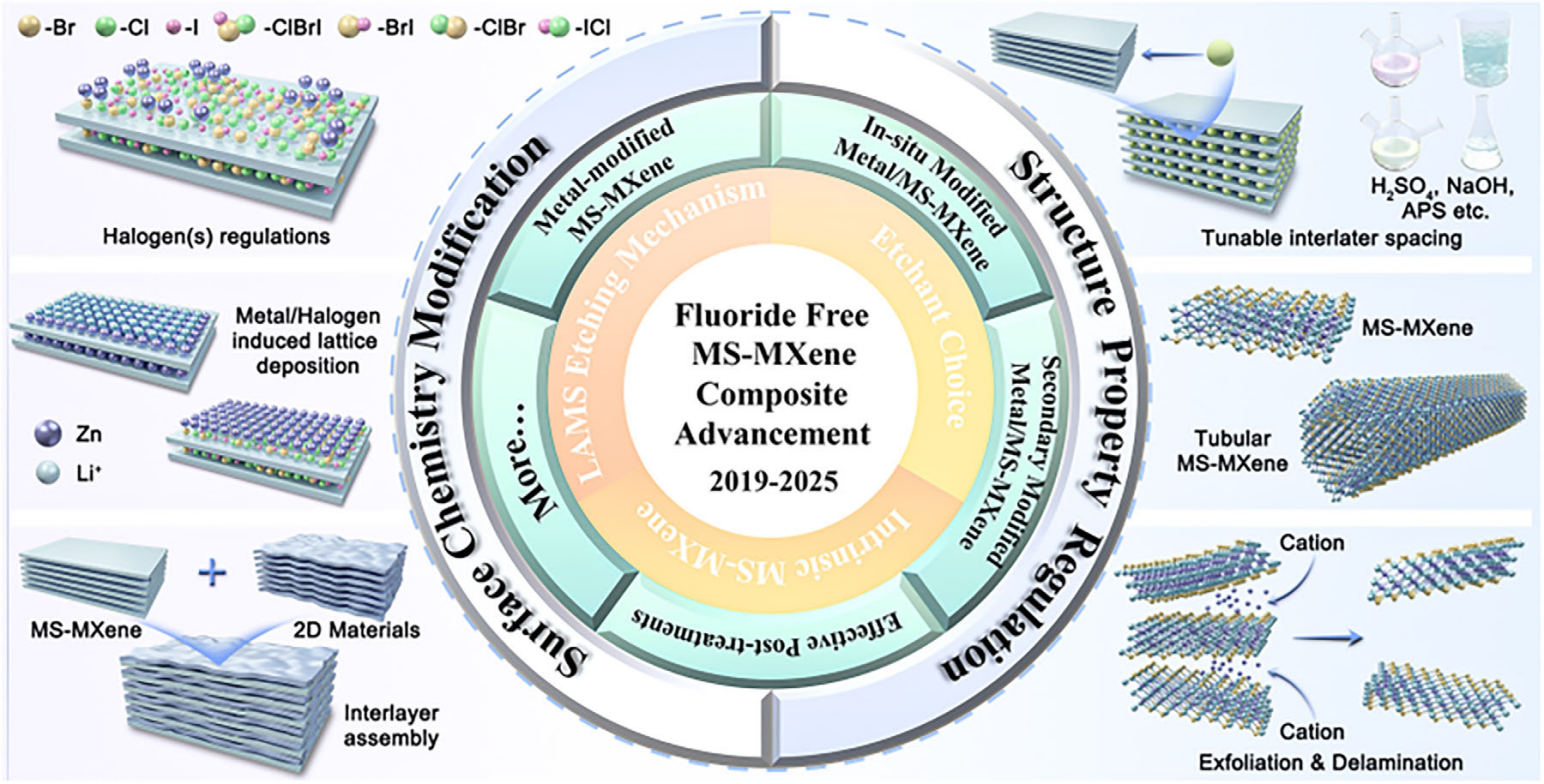
Tunable surface chemistry and structure–property regulation in fluoride‐free LAMS‐derived MXenes.

In contrast, Lewis acid molten salt (LAMS) etching typically produces MXenes with uniform halide terminations (MS‐MXenes), offering greater synthetic tunability than conventional fluoride‐based routes [9]. The diversity of halogens and Lewis salts, together with synthesis‐dependent modifications, significantly expands both the accessible MXene compositions and surface chemistries, leading to a substantially enlarged chemical and electronic design space defined by diverse halogen‐metal combinations [10]. In addition, the absence of polar terminations such as –O and –OH generally promotes more stable interlayer configurations [11]. Importantly, MXene surface chemistry can be modulated across multiple levels through the interplay of MAX precursors, etchant selection, reaction conditions, and post‐synthetic treatments (Figure 1). This high degree of synthetic flexibility gives rise to strongly coupled and complex relationships among synthesis pathways, surface chemistry, and electronic structure. Without such synthesis‐informed context, it remains challenging to parameterize the relevant chemical space, identify physically meaningful descriptors, and translate predictive insights into experimentally accessible design strategies [12].

Against this background, translating inverse design concepts to MXene materials is inherently challenging because their functionality is dictated not only by bulk composition, but more critically by synthesis‐dependent surface chemistry and interlayer environments [13]. As a result, the relationships among synthesis parameters, surface terminations, electronic structures, and performance are strongly coupled and nonlinear. Recent advances in artificial intelligence (AI) provide practical tools for addressing this problem [14]. In particular, synthesis conditions and surface states can be encoded into chemically meaningful representations through descriptor engineering, graph‐based representations, or spectroscopic features, thereby linking experimentally controllable variables with local electronic structure [15]. Machine learning (ML) architectures such as graph neural networks (GNNs) and attention‐based models can then learn the high‐dimensional mappings between surface chemistry, electronic structure, and functional performance [16]. On this basis, inverse design strategies, such as generative models [17], reinforcement learning [18], and active learning [19], can be used to search the synthesis space for target surface states and interfacial environments, rather than merely screening nominal compositions, thus enabling a more realistic route toward functional MXene discovery. Importantly, the success of such data‐driven frameworks also relies on scalable physics‐based data generation. High‐throughput density functional theory (DFT) calculations can systematically evaluate large numbers of surface configurations and reactive intermediates, forming the initial data foundation for data‐driven modeling [14]. In addition, active learning frameworks can iteratively couple ML predictions with targeted first‐principles modeling, selectively sampling the most informative surface configurations and thereby accelerating the exploration of the vast MXene surface chemistry space.

In this review, we highlight the emerging LAMS etching route that considerably expands the space of MXene surface chemistries. To bridge the knowledge gap between materials scientists and data scientists, we first discuss how MXene surface chemistries are formed, how LAMS etching broadens the accessible functionality, and how synthesis‐dependent surface chemistry governs performance. Building on this chemistry‐defined foundation, we then examine recent advances in descriptor design, scalable first‐principles modeling, and ML‐based inverse design which connect surface chemistry to accelerated functional MXene discovery. Finally, we discuss emerging high‐throughput and closed‐loop strategies from a realistic perspective, emphasizing current opportunities, data‐quality challenges, and the practical pathways needed to establish more reliable data‐driven workflows for molten‐salt‐derived MXene research.

## 2 MXene Synthesis Routes and Derived Surface Chemistry

MXene synthesis routes provide different levels of control over surface chemistry. Fluoride‐containing etching established the broad synthesis foundation of MXenes but usually introduces mixed terminations, while other non‐fluoride routes often remain constrained by etching efficiency, synthetic window, or limited termination tunability. LAMS is therefore highlighted here as a particularly attractive platform because its synthesis chemistry can be linked with relatively uniform terminations, highly tunable surface states, and coupled surface/interface regulation. In particular, Lewis acid‐derived metal species may be introduced or retained during etching, enabling surface metal incorporation and heterointerface formation to occur together with MXene synthesis. Although dedicated machine‐learning studies on LAMS‐derived MXenes remain limited, the following discussion identifies synthesis‐dependent surface and interfacial variables that may support future descriptor construction and data‐driven discovery.

### 2.1 Fluoride‐Containing Etching

MAX phases are layered M_n+1_AX_n_ compounds composed of transition metals (M), carbon and/or nitrogen (X), and A atoms (group IIIA to VIA elements) such as Al, Si, or Ga [20]. Their selective etching produces MXenes, M_n+1_X_n_T_x_, in which the exposed outer M layers are decorated by termination groups (T_x_), thereby defining their surface chemistry [21].

In fluoride‐containing etching, the A‐layer of MAX phases can be selectively removed because A atoms such as Al readily form stable soluble Al‐F species in acidic fluoride media, whereas the M‐X framework is relatively more resistant to dissolution due to its stronger bonding and kinetic stability under these conditions [22]. For Ti_3_AlC_2_, this drives the separation of Ti‐C slabs and the formation of layered MXenes. This chemistry can be achieved either by direct HF etching or indirect F‐containing routes with in situ HF generation (e.g., LiF/HCl or NH_4_HF_2_). Meanwhile, the freshly exposed MXene surfaces are terminated by –O, –OH, and –F species from the etching environment, producing mixed surface terminations [23].

Thus, the interplay between complexation by fluoride species and acid‐assisted dissolution is a key chemical determinant of MAX etching, as it governs the selective oxidation and solubilization of the A element and thus controls MXene formation. This principle can be extended to other MAX/MXene systems, where the etching chemistry must be matched to the bonding characteristics and solution chemistry of the specific A element [24]. From a data‐driven perspective, this suggests that the synthesizability‐constrained descriptors design can be informed by accessible thermodynamic quantities, such as phase formation energies, reaction free energies for A‐element removal, relative M─A/M─X bond energetics, and the formation energies of competing by‐products. Readers interested in more details of the MXene formation mechanism are referred to ref. [3].

### 2.2 Non‐Fluoride Etching

In LAMS etching, the A‐layer is removed through the redox‐driven extraction in halide melts [9]. Specifically, the Lewis acid withdraws electrons from the A element, enabling its oxidation and selective removal while preserving the M‐X framework. The feasibility of this process is largely governed by the redox potential matching between the target A element and the halide melt [25]. For instance, in chloride melts at 700 °C, Si^4+^/Si (−1.38 V vs Cl_2_/Cl^−^) is sufficiently negative relative to Cu^2+^/Cu (−0.43 V vs Cl_2_/Cl^−^), allowing CuCl_2_ to oxidize Si and thus drive the MAX/MXene transformation (Equations (1) and (2)). This redox‐driven mechanism is consistent with thermodynamic analysis based on Gibbs free energy differences between the Lewis acid melts and A‐site elements. A more general comparison of Gibbs free energies between Lewis acid chloride melts and the A site in MAX precursors at 700°C indicates that MAX phases (Ti_2_AlC, Ti_3_AlC_2_, Ti_3_AlCN, Nb_2_AlC, Ta_2_AlC, Ti_2_ZnC, and Ti_3_ZnC_2_) can be successfully etched into MS‐MXenes (Ti_2_CT_x_, Ti_3_C_2_T_x_, Ti_3_CNT_x_, Nb_2_CT_x_, Ta_2_CT_x_, Ti_2_CT_x_, Ti_3_C_2_T_x_) by using chloride‐based molten salts (CdCl_2_, FeCl_2_, CoCl_2_, CuCl_2_, AgCl, NiCl_2_). This mechanism guides the rational etchant choice mapping for diverse MAX precursors [9]. Importantly, non‐aqueous molten‐salt routes enable more uniform T_x_ coverage, which can be further substituted or decorated to achieve diverse functionalization [26].

(1) $$\begin{array}{c} Ti_{3}\mathrm{Si}C_{2}+2\mathrm{CuC}l_{2}\;\to \;Ti_{3}C_{2}+\mathrm{SiC}l_{4}\left(g\right)↑+2\mathrm{Cu} \end{array}$$

(2) $$\begin{array}{c} Ti_{3}C_{2}+\mathrm{CuC}l_{2}\;\to Ti_{3}C_{2}Cl_{2}+\mathrm{Cu} \end{array}$$

Moreover, the molar ratio of MAX to Lewis acid is also a decisive factor in successful etching to avoid the simple A‐site replacement [27]. In early LAMS etching studies, the process involves an intermediate A‐site replacement step prior to complete MXene formation [26]. As the first reported LAMS etching study, the first step is to use late transition metal zinc to occupy the “A” sites in the MAX phase structure, with the replacement reaction occurring between Zn in molten ZnCl_2_ and Al in MAX phase precursors (Ti_3_AlC_2_, Ti_2_AlC, Ti_2_AlN, and V_2_AlC). This process is facilitated by the formation of a liquid Al‐Zn eutectic within the A‐layer, which significantly enhances in‐plane atomic diffusion. When ZnCl_2_ was further employed, the Cl‐terminated MXenes (Ti_3_C_2_Cl_2_ and Ti_2_CCl_2_) could be successfully synthesized. Within the MAX phase, the formation of a liquid Al‐Zn eutectic within the two‐dimensional A sublayer, together with molten ZnCl_2_, serves to markedly accelerate the in‐plane diffusion of Al and Zn atoms, thereby facilitating the elemental exchange process at 550 °C. Thermodynamically, the Al‐Zn system forms a eutectic alloy at ∼385 °C, well below the etching temperature [28]. The calculated phase diagram at 550 °C indicates that Ti_3_ZnC_2_ was thermodynamically competitive with Ti‐Zn alloys and should be stable at equilibrium under these conditions. The A‐site replacement strategy exploited the low‐temperature diffusion of targeted elements while avoiding the formation of competing M‐A alloys. As the ZnCl_2_ content increased, the product evolved progressively from Ti_3_ZnC_2_ to Ti_3_C_2_Cl_2_, which can be attributed to the increased Lewis acidity and redox driving force of the molten salt system [29].

**FIGURE 2:**
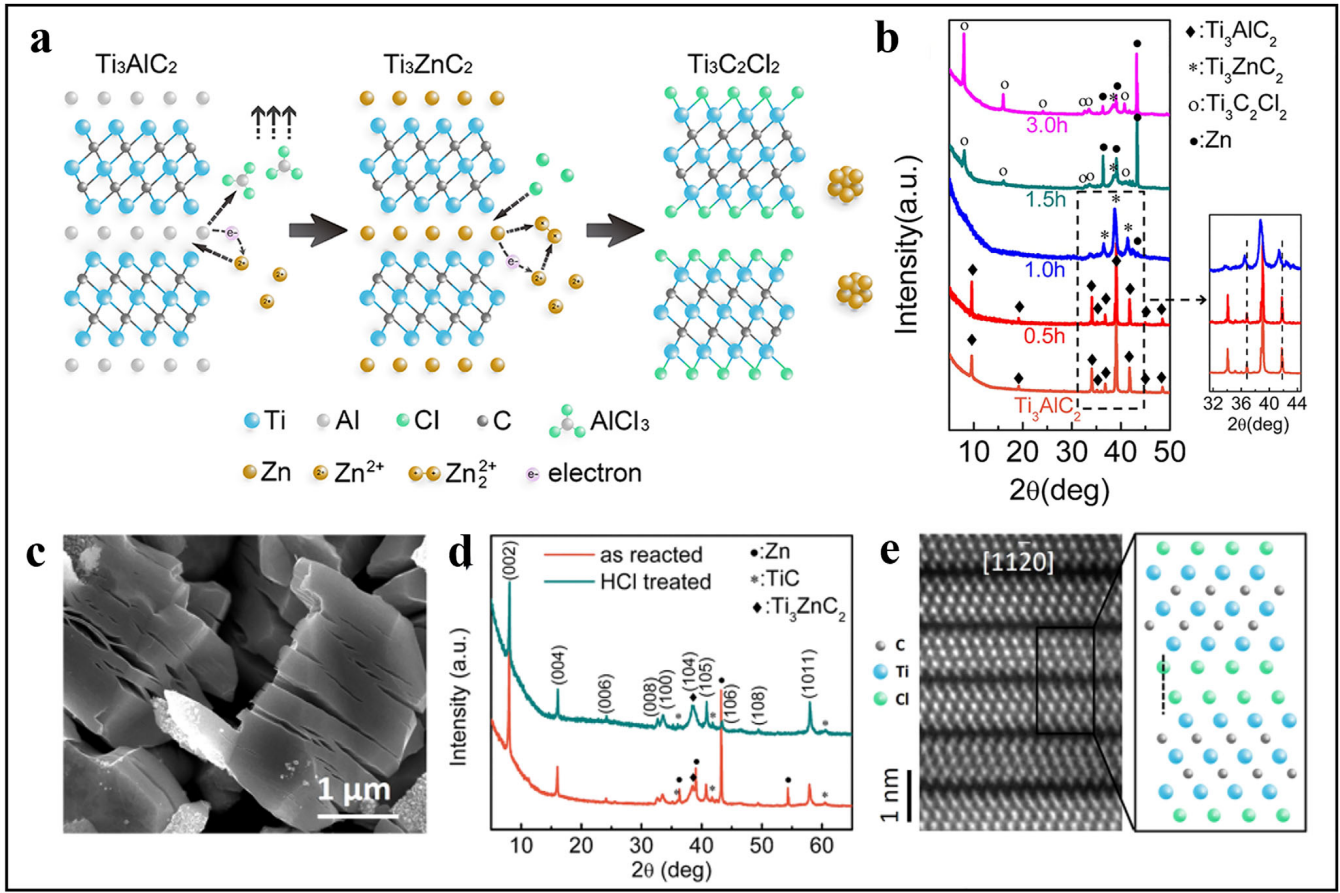
(a) Mechanism of Lewis acid melt replacement. (b) XRD patterns of samples obtained at Ti_3_AlC_2_/ZnCl_2_ = 1:6 with varied reaction times, showing the phase evolution from Ti_3_AlC_2_ to Ti_3_ZnC_2_ and finally to Ti_3_C_2_Cl_2_. (c) SEM image of Ti_3_C_2_Cl_2_. (d) XRD patterns of Ti_3_C_2_Cl_2_ with/without HCl treatment. (e) HRTEM image and corresponding structural model of Ti_3_C_2_Cl_2_ [26]. Reproduced with permission [26]. Copyright 2019, American Chemical Society.

The etching mechanism [26], exemplified by Ti_3_ZnC_2_ and depicted in Figure 2a, is proposed as follows:

(3) $$\begin{array}{c} Ti_{3}\mathrm{Al}C_{2}+1.5\mathrm{ZnC}l_{2}=Ti_{3}\mathrm{Zn}C_{2}+0.5\mathrm{Zn}+\mathrm{AlC}l_{3}↑ \end{array}$$

The reaction (3) can be decomposed into two sub‐reactions, namely (3.1) and (3.2):

(3.1) $$\begin{array}{c} Ti_{3}\mathrm{Al}C_{2}+1.5\mathrm{ZnC}l_{2}=Ti_{3}C_{2}+1.5\mathrm{Zn}+\mathrm{AlC}l_{3}↑ \end{array}$$

(3.2) $$\begin{array}{c} Ti_{3}C_{2}+\;\mathrm{Zn}=Ti_{3}\mathrm{Zn}C_{2} \end{array}$$

(4) $$\begin{array}{c} Ti_{3}\mathrm{Zn}C_{2}+\mathrm{ZnC}l_{2}=Ti_{3}C_{2}Cl_{2}+2\mathrm{Zn} \end{array}$$

Reaction (4) can be divided into sub reactions (4.1), (4.2), and (4.3):

(4.1) $$\begin{array}{c} Ti_{3}\mathrm{Zn}C_{2}+\;Zn^{2+}=Ti_{3}C_{2}+\;Z{n_{2}}^{2+} \end{array}$$

(4.2) $$\begin{array}{c} Ti_{3}C_{2}+2Cl^{-}=Ti_{3}C_{2}Cl_{2}+2e^{-} \end{array}$$

(4.3) $$\begin{array}{c} Z{n_{2}}^{2+}+2e^{-}=2\mathrm{Zn} \end{array}$$

The phase evolution of Ti_3_AlC_2_ to Ti_3_ZnC_2_ to Ti_3_C_2_Cl_2_ (Figure 2b) was revealed with increasing reaction time from 0.5 h to 3.0 h with 0.5 h intervals at 550 °C. The results systematically demonstrate that the ZnCl_2_ etchant transforms Ti_3_AlC_2_ into Ti_3_ZnC_2_ within 1.0 h. After 1.5 h, with non‐basal‐plane peaks of Ti_3_ZnC_2_ vanishing and the (000*l*) reflections of Ti_3_C_2_Cl_2_ appearing, the Ti_3_ZnC_2_ was transformed into Ti_3_C_2_Cl_2_. It should be noted that the 3.0 h sample displays highly crystallized Ti_3_C_2_Cl_2_ and the sharp peaks of free Zn, which illustrates that the as‐obtained product is a combination of Ti_3_C_2_Cl_2_ and Zn atoms. The morphology of Ti_3_C_2_Cl_2_ (Figure 2c) shows a densely stacked layered structure with Zn spheres on the surface. The as‐obtained product was also treated with HCl to remove surface Zn (Figure 2d). The orderly terminated Cl atoms are also well aligned along the (000*l*) plane (Figure 2e).

In recent years, molten‐salt and halogen‐based etching routes have attracted increasing attention. First, these routes can deliver MXenes with surface terminations far beyond those typically accessible from HF‐based etching, including –Cl, –Br, –I, –NH, –S, –Se, –Te, and vacancy terminations [30]. Beyond providing a greener fluoride‐free etching strategy, these routes also expand the accessible MXene family to compositions that are challenging to achieve by F‐containing methods, especially the nitride MXenes. The molten salt shielding strategy (MS^3^) was also developed to utilize a eutectic molten‐salt system, where a reduction of the overall energetic demand of LAMS etching can be achieved [27]. Although such systems enable open‐air synthesis by protecting MXenes from high temperature oxidation, safe implementation still requires compatible ceramic crucibles, controlled heating conditions, and careful salt containment. Within this controlled processing window, the etching scale of Ti_3_AlC_2_ has been enlarged to 60 g, yielding Ti_3_C_2_T_x_ MS‐MXene with a yield ratio of 72.3%. This result highlights the practical potential of LAMS‐etching for efficient and scalable MXene production.

Moreover, LAMS‐derived MXenes offer a distinctive and highly tunable chemical‐structural space rather than merely an alternative synthetic paradigm. Their well‐defined halogen‐rich surface environment has enabled systematic regulation of multi‐halide coverage [31], surface chemistry‐induced lattice matching [32], and interfacial coupling with other two‐dimensional (2D) materials [33], highlighting the strong sensitivity of interfacial behavior to surface state design [34]. Meanwhile, their structural degrees of freedom, including interlayer spacing, morphology, and exfoliation or delamination behavior, provide additional handles for tuning the ion transport, interfacial accessibility, and assembly pathways [35]. Taken together, these features indicate that LAMS MXenes constitute a unique platform in which the surface chemistry, structural regulation and interfacial interactions can be coupled in a controllable manner.

Beyond fluoride‐containing etching and LAMS routes, several alternative strategies such as electrochemical etching [36], alkali etching [37], chemical vapor deposition (CVD) growth [38], and thermal reduction [39] have also been explored for MXene synthesis. From the perspective of accessible surface chemistry, these methods generally lead to a relatively narrow range of surface states. Electrochemical and alkali routes mainly produce –O and –OH terminated surfaces, while CVD can yield nearly bare MXene surfaces, and UV‐induced conversion or thermal reduction tends to generate O‐rich terminations.

In particular, the simple and fluorine‐free surfaces can be valuable for probing the intrinsic surface reactivity, minimizing F‐related interference. However, compared with fluoride‐based methods, these routes offer lower etching efficiency or narrower synthetic windows, and compared with LAMS, they still lack a degree of uniformity and functionalization space [30]. For example, electrochemical etching is attractive as a greener fluoride‐free route, yet the limited accessibility of electrolyte species to the MAX interlayers often restricts efficient etching to the surface region and lowers the yield unless intercalants are introduced. Meanwhile, the possible presence of residual salts in LAMS etching that are mostly soluble can be removed by deionized water washing, which preserves a relatively clean surface environment. The site‐selective redox reactions also help generate uniform and controllable surface terminations. These features make LAMS etching a direct synthesis route to defined surface states, providing the reproducible chemical basis for the surface engineering strategies discussed in the following section.

This mechanistic picture also clarifies which synthesis information should be recorded for later modeling. LAMS etching can be represented by controllable synthesis inputs, including MAX precursor, A‐site element, Lewis acid salt, eutectic medium, salt‐to‐precursor ratio, reaction temperature, reaction time, atmosphere, and washing protocol. These inputs define the chemical environment for A‐site extraction and surface termination formation. Their experimental outputs can be recorded as phase conversion, yield, interlayer spacing, termination identity, residual metal content, metal distribution, morphology, and flake size. These experimental variables are more useful for data‐driven analysis when they are paired with physically meaningful calculated descriptors. Redox compatibility, reaction free energy for A‐site extraction, and competing phase stability can describe whether a given precursor‐salt combination is likely to form an MXene phase. Surface binding energy can further describe whether the resulting surface state is thermodynamically reasonable.

Among these variables, precursor type, Lewis acid salt, salt‐to‐precursor ratio, temperature, reaction time, atmosphere, and washing protocol are directly recordable synthesis inputs. Phase conversion, yield, interlayer spacing, morphology, and residual metal content can be quantified with relatively high confidence through XRD, microscopy, and elemental analysis. In contrast, local Lewis acidity in molten salts, transient A‐site extraction intermediates, salt activity, and washing‐induced termination exchange remain difficult to measure directly and should be represented through calculated descriptors or carefully defined experimental proxies. In this way, LAMS synthesis can be converted from a descriptive experimental procedure into the first dataset layer linking reaction conditions, surface state formation, and model‐guided MXene discovery.

## 3 Tunable Surface Chemistry in LAMS‐Derived MXenes

Through controllable termination chemistry, surface metal incorporation, and diverse further modification strategies, LAMS‐derived MXenes open a broader design space for surface chemistries and provide a more efficient synthesis platform for function‐oriented engineering [2]. These surface states should be regarded as functional variables because they directly influence local electronic structure, charge redistribution, ion transport, adsorption behavior, and interfacial stability. Experimentally measurable features, such as termination identity, termination coverage, interlayer spacing, residual metal species, and local coordination environment, can further be translated into descriptors for data‐driven modeling.

This expanded design space also requires careful control during post‐synthetic processing. Washing and delamination can further alter the surface chemistry of LAMS‐derived MXenes, and these changes often show strong time‐dependent features, especially through partial replacement of halogen terminations, oxidation, hydrolysis, or intercalation‐related structural changes. Therefore, post‐treatment should be regarded as an active stage of surface‐chemistry regulation. A clearer understanding of these processing‐induced changes is essential for improving reproducibility and establishing reliable links between synthesis conditions, surface descriptors, and final material functions.

In descriptor construction, these surface‐regulation strategies can be viewed as a hierarchy with increasing chemical complexity. Termination engineering provides the most fundamental descriptor layer because termination identity, coverage, and interlayer response are directly linked to the initial LAMS etching chemistry. Surface metal incorporation then expands this descriptor space by introducing matrix‐termination‐metal coupling. In situ and ex situ modification further add pathway‐dependent interface descriptors, while post‐synthetic modification introduces treatment‐dependent and time‐dependent surface evolution variables. Therefore, the descriptor system should evolve with the synthetic strategy, rather than treating all LAMS‐derived MXenes with the same composition‐based representation.

### 3.1 Surface Termination Engineering

Surface termination is the most fundamental feature of MXenes, as it directly regulates their intrinsic properties. The mixed terminations derived from fluoride‐containing etching strategies have been well studied. However, the coverage of mixed terminations is difficult to reproduce with high experimental consistency. The LAMS etching strategy brings MXene surface regulation into a more uniform and controllable chemical space [4]. Table 1 summarizes the representative surface terminations and the respective functional roles.

**TABLE 1:** Summary of representative MXene functionalization by termination.

MXene	Precursor/Etchant	Surface termination	Functional role	Application	Refs.
Ti_3_C_2_T_x_	Ti_3_AlC_2_, LiF/HCl	–O, –OH, –F	Macroporous ion‐accessible architecture	Non‐aqueous Li‐ion battery	[40]
Ti_3_C_2_T_x_	Ti_3_AlC_2_, LiF/HCl	–O, –OH, –F	Vertically aligned liquid‐crystalline assembly	Supercapacitor	[41]
Ti_3_C_2_Cl_2_	Ti_3_AlC_2_; CuCl_2_	–Cl	Uniform –Cl coverage for charge transfer kinetics	Non‐aqueous Li‐ion battery	[9]
Ti_3_C_2_Cl_2_	Ti, Al, C powders; CuCl_2_	–Cl	Uniform –Cl coverage for charge transfer kinetics	Non‐aqueous Li‐ion battery	[42]
Ti_3_C_2_X_2_/Ti_3_C_2_XY/Ti_3_C_2_XYZ (X, Y, Z = Cl, Br, I)	Ti_3_AlC_2_; CuI/CuBr/CuCl_2_	–I, –Br, –Cl; binary and ternary halogens	Highly reversible redox behavior of surficial halogens	Aqueous Zn‐ion battery	[31]
I‐Ti_3_C_2_	Ti_3_AlC_2_; CuI	–I	Capacitive‐controlled redox behavior of interlayer I_2_	Supercapacitor	[43]

The most basic modification is the direct construction of Cl‐terminated MS‐MXenes, where the halogen‐covered surface provides polarizable yet weakly coordinating anion sites that can compete with solvent molecules without excessively immobilizing Li^+^, thereby creating a more favorable interface for non‐aqueous Li‐ion storage [9]. Along this line, we first highlight an efficient coupling strategy that achieves a direct conversion from elemental precursors to Cl‐covered MS‐MXenes through LAMS chemistry [42]. The molten salt acts simultaneously as the reaction medium and oxidation shield at high temperature, offering a more cost‐effective alternative to pre‐synthesized MAX precursors. This strategy apparently can be extended to other MXene synthesis.

**FIGURE 3:**
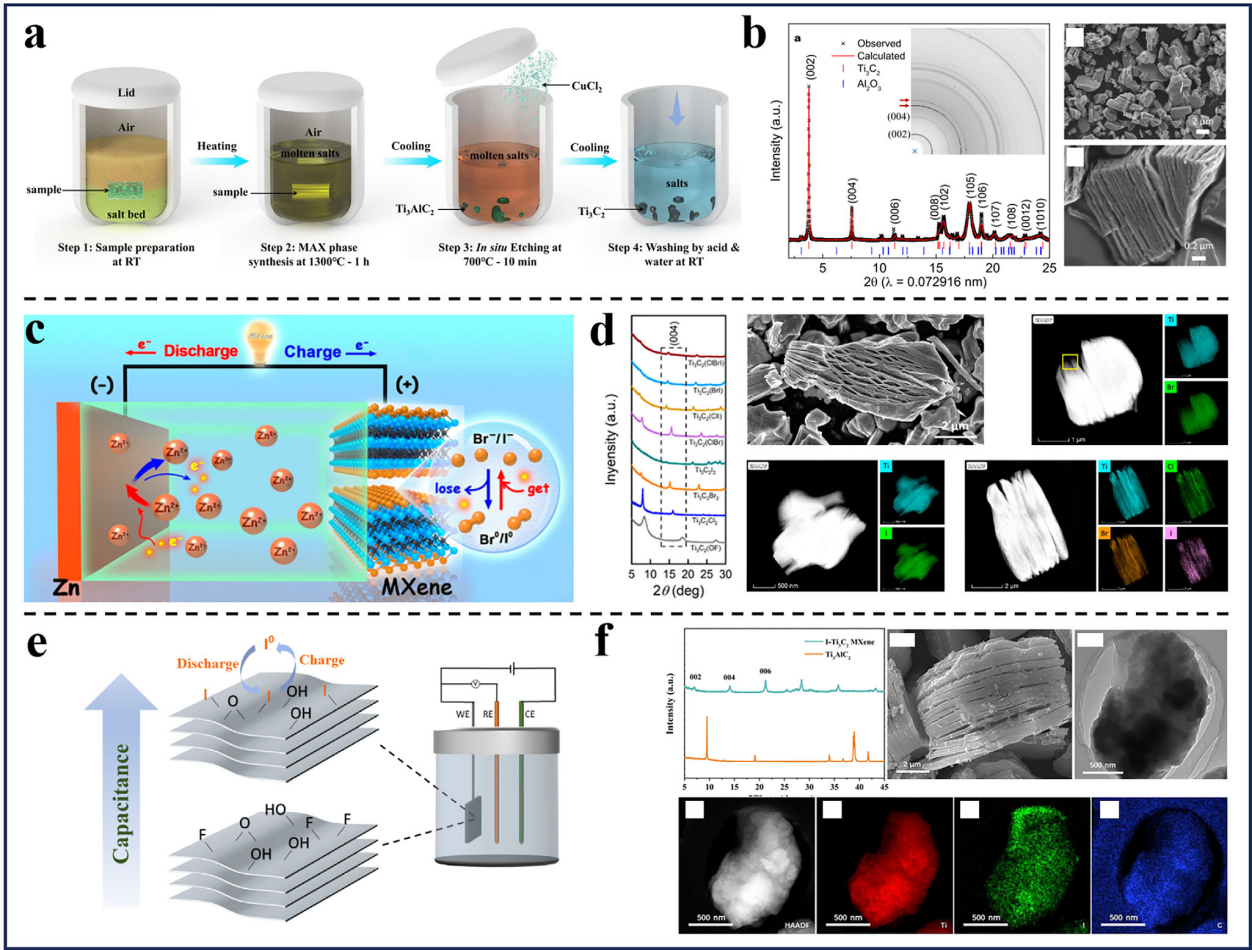
(a) Schematic diagram of the one‐pot synthesis of Ti_3_C_2_T_x_ MXene in open air with elemental Ti, Al, and C powders as starting materials. (b) 1D and 2D synchrotron X‐ray (λ = 0.072916 nm) diffraction (SXRD) patterns, Rietveld refinement, and SEM images of Ti_3_C_2_T_x_ prepared by etching at 700°C for 10 min [42]. Reproduced with permission [42]. Copyright 2021, Springer Nature. (c) Sketch of halogen terminated Ti_3_C_2_ in a zinc‐ion battery. (d) XRD patterns of halogen terminated MS‐MXenes, together with SEM images, HAADF‐STEM images, and EDS elemental mappings of Ti_3_C_2_Br_2_, Ti_3_C_2_I_2_, and Ti_3_C_2_(ClBrI) [31]. Reproduced with permission [31]. Copyright 2021, American chemical Society. (e) Scheme of iodine‐terminated MXene for supercapacitor. (f) XRD patterns, SEM images, and corresponding elemental mappings of I‐Ti_3_C_2_ MXene [43]. Reproduced with permission [43]. Copyright 2022, Elsevier.

In 2019 Dash et al. first reported a MS^3^ strategy [44] for the preparation of MAX phases in open air at temperatures beyond 1000°C. Roy et al. proposed eutectic salts of NaCl and KCl for the MS^3^‐derived V_2_AlC and Ti_2_AlN [45]. Thus, Lin et al. further used the elemental powders, eutectic salts (NaCl and KCl) to first synthesized the Ti_3_AlC_2_ MAX precursor [42]. When the temperature cooled back to 700°C, CuCl_2_ was subsequently added, and the LAMS etching could be completed within only 10 min (Figure 3a). The total processing time is less than 8 h with MS^3^ method coupled, considerably increasing the synthesis efficiency and economic viability of MXene. The interlayer spacing of *d* = 1.1107 nm (*a* = 0.318 nm and *c* = 2.213 nm) can be revealed by XRD results (Figure 3b). However, in this study, the differences in etching time were not reflected in XRD patterns, which show almost complete overlap over the full 2θ range. The SEM images clearly show the multilayered and uniform structure of MS^3^‐Ti_3_C_2_T_x_, with an average size below 5 µm. The obtained MS^3^‐Ti_3_C_2_T_x_ were further applied for Li‐ion storage. In 2025, the carbon precursor was extended to carbon fiber by Filipa M. Oliveira et al., where the tubular MS‐Ti_3_C_2_T_x_ was also achieved with fine morphological control by MS^3^ method in open air. However, compared with traditional 2D MXene, the tubular structure demonstrated irreversible electrochemical behavior, and therefore delivered lower Li‐ion capacity and stability [46].

The halogen‐terminated Ti_3_C_2_ MXene with unary, binary and ternary terminations (e.g., –Cl, –Br, –I, –BrI, and –ClBrI), was systematically examined for aqueous zinc‐ion battery, disclosing the effect of the termination chemistry‐induced redox behavior on MS‐MXene (Figure 3c). Structurally, the shift of the (0004) diffraction peak with different surface terminations reflects variations in the *c*‐lattice parameter of MS‐MXene (Figure 3d), with major reflections corresponding to *c* values of 23.33 Å for Ti_3_C_2_Br_2_ and 25.00 Å for Ti_3_C_2_I_2_. The increased interlayer spacing in these materials can be attributed to the larger atomic radii of the halogens. Clearly, it is both effective and reasonable to modify surface terminations through Lewis acid etching, thereby regulating the *c*‐lattice parameter of MXene. Sequentially, it is noteworthy that the halogen‐terminated MS‐MXenes, for instance the SEM image of Ti_3_C_2_Br_2_ (Figure 3d), exhibit stronger van der Waals interactions between individual nanosheets. In this work, halogen terminations were introduced by employing Lewis acids such as CuCl_2_, CuBr_2_, or CuI, either individually or in specific binary/ternary mixtures, followed by a 7 h heat treatment at 700°C, and were subsequently washed with deionized water and ammonium persulfate (APS, (NH_4_)_2_S_2_O_8_). Furthermore, the specific iodine‐terminated Ti_3_C_2_ MXene was evaluated as a supercapacitor electrode [43]. The highly reversible redox behavior of surface iodine termination governs the enhanced capacitance, compared with the mixed terminations induced by F‐containing routes (Figure 3e). The EDS mappings (Figure 3f) verify the uniform distribution of halogen terminations on Ti_3_C_2_ nanosheets.

**FIGURE 4:**
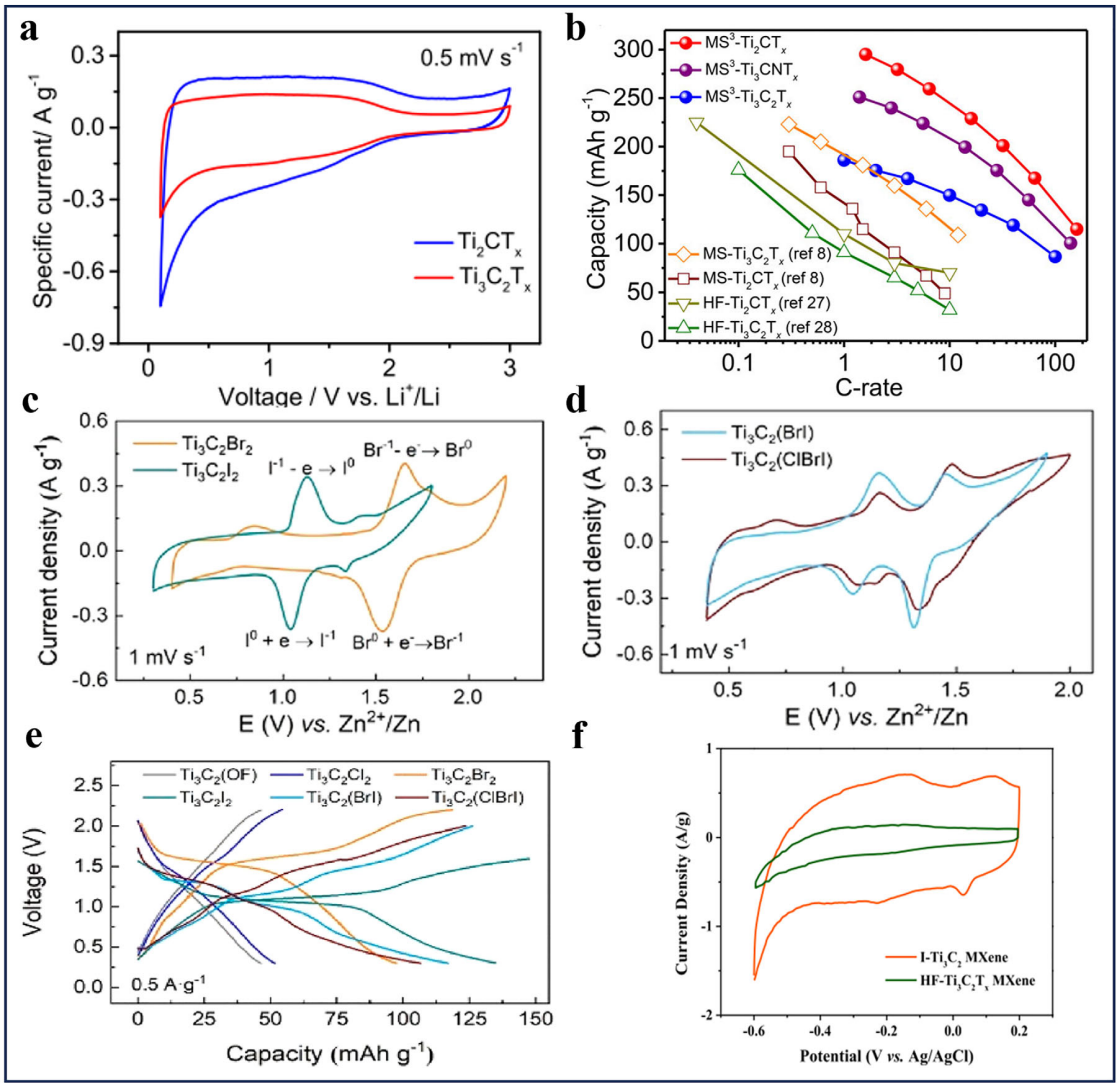
a) CV profiles of Ti_3_C_2_T_x_ and Ti_2_CT_x_ MXene at 0.5 mV s^−1^. b) Specific capacities of MS^3^‐MXenes, MS‐MXenes, and HF‐MXenes at different C‐rate [42]. Reproduced with permission [42]. Copyright 2021, Springer Nature. c) CV profiles of Ti_3_C_2_Br_2_ and Ti_3_C_2_I_2_ at 1 mV s^−1^. d) CV profiles of Ti_3_C_2_(BrI) and Ti_3_C_2_(ClBrI) at 1 mV s^−1^. e) Typical GCD curves of Ti_3_C_2_ MXenes with different terminals at the current density of 0.5 A g^−1^ [31]. Reproduced with permission [31]. Copyright 2021, American chemical Society. f) CV profiles of I‐Ti_3_C_2_ MXene and HF‐Ti_3_C_2_T_x_ MXene at 5 mV s^−1^ [43]. Reproduced with permission [43]. Copyright 2022, Elsevier.

The surface chemistry of MS‐MXenes, especially the halogen‐terminated features, directly govern the derived electrochemical behavior, and have been translated into performance in energy‐storage systems, including Li‐ion batteries [42], zinc‐ion batteries [31], and supercapacitors [43]. The Ti_3_C_2_T_x_ and Ti_2_CT_x_ MXene synthesized through the MS^3^ strategy with MAX phase elemental precursors demonstrated rectangular and highly symmetric cyclic voltammetry (CV) profiles (Figure 4a) within a potential range from 0.1 to 2 V without the presence of visible redox peaks, which verifies the pseudocapacitive Li‐ion storage ability of MS‐MXene [47]. The comparison of specific capacity at different C rates between HF‐MXenes and MS^3^‐MXenes (Figure 4b) suggests that MS^3^‐MXenes possess higher capacity and further improved power performance for Li‐ion storage [48]. The results can be traced back to the synthetic differences, specifically the avoidance of high‐temperature oxidation enabled by the MS^3^ method. Thus, MS^3^ method can avoid the high‐temperature oxidation, which helps retain the structural integrity and electronic conductivity of MXenes and facilitates faster charge transport kinetics and more efficient Li^+^ storage. For halogen‐terminated MS‐MXenes, Ti_3_C_2_Br_2_ and Ti_3_C_2_I_2_ both show distinct redox peaks: 1.55/1.65 V for Ti_3_C_2_Br_2_ and 1.05/1.15 V for Ti_3_C_2_I_2_ (vs Zn/Zn^2+^), which also demonstrates the strong coupling of Br‐ or I‐based halogen terminations with MS‐MXene. The behavior is therefore totally different from Ti_3_C_2_(OF) and Ti_3_C_2_Cl_2_ reported elsewhere [49]. Also, the Ti_3_C_2_I_2_ offers a more favorable electrochemical window for avoiding competing hydrogen evolution [50].

The absence of differences between the CV curves of Ti_3_C_2_(ClBrI) and Ti_3_C_2_(BrI) provides clear evidence that the Cl terminations make little contribution to electrochemical activity under the present conditions. Li et al. also proposed that the larger radius of I^−^ than Br^−^ could be the reason for the reduced redox potential of Br^−^/Br^0^ in multi‐halogen‐terminated MXenes, owing to the distorted octahedral structure [TiC_3_Br_n_I_3−n_]. The galvanostatic charge‐discharge (GCD) profiles (Figure 4e) show Ti_3_C_2_Br_2_ and Ti_3_C_2_I_2_ characterized by plain discharge platforms at 1.6 and 1.1 V with the capacities of 97.6 and 135 mAh g^−1^, respectively. The total capacities of Ti_3_C_2_Br_2_ and Ti_3_C_2_I_2_ consist of two components. One arises from pseudocapacitance, which is comparable to that of Ti_3_C_2_Cl_2_ and Ti_3_C_2_(OF). The other, accounting for approximately 45% of the overall capacity, originates from the discharge plateau [31]. The higher pseudocapacitance of Ti_3_C_2_Cl_2_ can be ascribed to the wider interlayer spacing, which provides additional pathways for ion transport, compared with that of Ti_3_C_2_Br_2_. The sharp redox characteristic peaks at −0.15/−0.25 V in the CV comparison (Figure 4f) between I‐Ti_3_C_2_ MXene and HF‐Ti_3_C_2_ MXene exhibit that the reversible conversion of I^−^/I° contributes to improving the specific capacitance. The shapes of the CV plots feature the characteristics of pseudocapacitive materials [51]. The I‐terminated MS‐MXene is subsequently treated by a lyophilization process for full exfoliation. This greatly improved its electrochemical performance compared with raw I‐terminated MS‐MXene mentioned before, ascribed to the structural maintenance.

These examples establish termination chemistry as the first level of surface state control in LAMS‐derived MXenes, where termination identity and coverage regulate interfacial charge distribution, ion adsorption, redox activity, and interlayer transport. From a data‐driven perspective, termination engineering is the most tractable surface chemistry level for LAMS‐derived MXenes because the chemical variables are relatively well defined. Key dataset entries include precursor type, Lewis acid salt, halogen identity, halogen combination, reaction temperature, reaction time, washing condition, and measured termination coverage. These entries can be linked to experimentally accessible outputs such as *c*‐lattice parameter, interlayer spacing, redox potential, charge‐storage capacity, and cycling stability. They can also be paired with calculated descriptors, including surface binding energy, work function, charge distribution, ion adsorption energy, and diffusion barrier. Compared with metal‐containing heterostructures, termination‐engineered LAMS‐derived MXenes provide a cleaner starting point for model construction because their surface chemistry is more uniform and compositionally better constrained.

For termination engineering, the data reliability is not uniform across all surface descriptors. Halogen identity, halogen combination, lattice parameter, and interlayer spacing are relatively robust because they can be supported by elemental analysis and XRD. Termination coverage is more informative for modeling but less straightforward to quantify. XPS fitting, elemental balance, and DFT‐assisted interpretation are commonly used, while STEM‐based elemental mapping can support the spatial uniformity of heavier halogen terminations but is generally insufficient for determining absolute coverage. Local termination ordering, vacancy distribution, and termination exchange during washing remain difficult to quantify directly, and are better represented through calculated surface configurations or descriptors stated with uncertainty.

### 3.2 LAMS‐Derived Surface Metal Incorporation

Beyond termination, the Lewis acid derived metal species can also be well retained and serve as a surface‐regulating component (Table 2). We ascribe such functionality to incorporation of surface metal, because it extends the formation of MS‐MXene to a broader heterostructure design strategy. The surface metal incorporation is discussed here as an extension of LAMS‐derived surface chemistry, not MXene composites. The key distinction is that the interface is not constructed on an inert MXene support. Instead, LAMS‐derived terminations and Lewis acid‐derived metal species provide chemically defined surface states that can guide interfacial growth, anchoring, or conversion.

**TABLE 2:** Summary of MXene functionalization strategies beyond termination.

Type	Material	Strategy	Functional role	Application	Refs.
LAMS‐derived surface metal incorporation	Ti_3_C_2_‐Cu/Co	Retention of Lewis acid‐derived metal species	Surface‐controlled redox behavior of metal site by –O bridge	Supercapacitor	[52]
	Ti_3_C_2_T_x_/Sn	Retention of Lewis acid‐derived metal species	Enlarged and stabilized interlayer active spacing	Non‐aqueous Li‐ion battery	[53]
Surface directed heterostructure engineering (in situ)	MX‐CoS_2_, N‐MX‐CoS_2_	Sulfur/Thiourea powder for in situ sulfidation	Formation of MXene‐metal sulfide heterostructures with enhanced redox activity	Lithium‐sulfur battery	[54]
	Ti_3_C_2_T_x_/FeS_2_, Ti_3_C_2_T_x_/CoS_y_, Ti_3_C_2_T_x_/NiS_y_	Thiourea powder for in situ sulfidation	Surface‐anchored metal sulfides enabling improved ion storage and charge transfer	Sodium‐ion battery	[55]
	Ti_3_C_2_T_x_/CuS	Thiourea powder for in situ sulfidation	Formation of conductive sulfide domains improving electrochemical kinetics	Sodium‐ion battery	[56]
	Co/Co‐N‐C@Ti_3_C_2_Cl_2_	ZIF‐67 Co‐pyrolysis	Construction of Co‐N‐C active sites for enhanced ORR activity	ORR electrocatalyst	[57]
Surface directed heterostructure engineering (ex situ)	Zn‐Ti_3_C_2_Cl_x_‐Li	Li‐nucleation with immobilized surficial Zinc	Regulated Li nucleation and suppressed dendrite growth	Li‐ion Battery	[58]
	Ti_3_C_2_Cl_2_‐Zn	Zn deposition with halogen regulated lattice matching	Uniform Zn deposition and improved interfacial stability	Zinc‐ion Battery	[32]
	HD‐Fe‐Ti_3_C_2_T_x_	HCl‐etching for highly dispersed surficial Fe atoms	Formation of isolated Fe sites enhancing catalytic activity	NRR Electrocatalyst	[59]
	QD‐Ti_3_C_2_Cl_2_@NiAl‐LDHs	Exfoliation coupled with electrostatic interstratification‐assembly	Hierarchical heterostructures improving ion accessibility	Supercapacitor	[60]
	Au/Pt/Ti_3_C_2_Cl_2_	Self‐reduction with utilization of Ti_3_C_2_Cl_2_ as reductant	Formation of noble metal nanoparticles with strong interfacial coupling	H_2_O_2_ detector	[61]
	Bi_12_O_17_Cl_2_/Ti_3_C_2_	Chlorine termination regulated growth	Enhanced interfacial charge transfer for photocatalysis	Photodegradation of methyl blue	[62]
Post‐synthetic modification	SACs‐Cu‐Ti_3_C_2_T_x_	Precursor‐encoded single‐atom decoration	Atomically dispersed Cu sites; unsaturated coordination; enhanced electron transfer	CO_2_ electroreduction	[63]
	Ti_3_C_2_T_x_/Co hybrid	Acid‐regulated surface metal speciation	Tuned balance of lattice‐associated and interlayer Co; optimized active‐species distribution	HER	[64]
	TBA^+^‐delaminated Ti_3_C_2_Cl_2_	Ion exchange‐assisted delamination	Improved wettability, colloidal stability, and active‐surface utilization	Li‐ion battery anode	[65]
	Electrochemically delaminated Ti_3_C_2_Cl_2_	Electrochemical swelling delamination	Few‐layer dispersion with preserved halogen terminations; high delamination yield	Tribovoltaic nanogenerator	[66]
	LiCl‐delaminated Ti_3_C_2_Cl_2_	LiCl‐assisted conductive delamination	Stable colloids; preserved Cl terminations; high film conductivity	Conductive film electronics	[67]
	Mesoporous Ti_3_C_2_Cl_2_	Etching‐coupled porosity engineering	Mesoporous Ti_3_C_2_Cl_2_; enlarged surface area; enhanced ion accessibility	Dual‐ion battery anode	[68]
	Ti_3_C_2_X_2_ (X = S, Te, etc.)	Molten‐salt termination substitution	Expanded termination chemistry; reactive transformable surface platform	Surface chemistry engineering	[30]
	Ti_3_C_2_T_x_	Aqueous termination transformation	Shifted termination population; APS‐induced O enrichment; NaOH‐induced TiO_2_ side product	Li‐ion storage	[69]
	Ti_3_C_2_Cl_2_‐CsPbBr_3_	Termination‐enabled interfacial regulation	Strain relaxation; enlarged grains; compact grain boundaries	Perovskite solar cells	[70]

**FIGURE 5:**
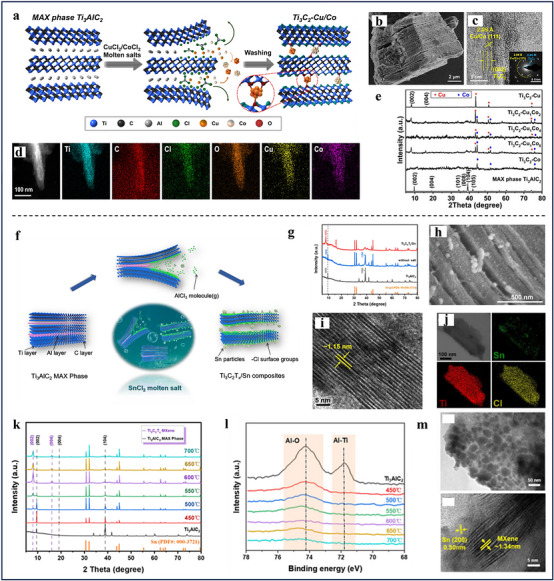
(a) Scheme of preparation of the synthesis of Ti_3_C_2_‐Cu/Co hybrids. (b) SEM image, (c) HRTEM image, and (d) HAADF‐STEM image and EDS mapping of Ti_3_C_2_‐Cu_1_Co_8_. (e) XRD patterns of Ti_3_AlC_2_ and Ti_3_C_2_‐Cu/Co hybrids with different Cu/Co compositions [52]. Reproduced with permission [52]. Copyright 2021, John Wiley and Sons. (f) Schematic illustration of the synthesis of Ti_3_C_2_/Sn composites by SnCl_2_. (g) XRD patterns of Ti_3_C_2_T_x_/Sn composites, Ti_3_C_2_T_x_/Sn without NaCl/KCl salt, and pristine Ti_3_AlC_2_ MAX phase powders. (h) SEM image, (i) HRTEM image, and (j) STEM image and the corresponding EDS mapping images of Ti_3_C_2_T_x_/Sn composites. (k) XRD patterns of pristine Ti_3_AlC_2_ and reaction products obtained at temperatures ranging from 450 to 700°C. (l) The variation trend of XPS spectra of Al 2p energy level. m) TEM and HRTEM images of Ti_3_C_2_T_x_/Sn composite anode after long cycles at 200 mA g^−1^ [53]. Reproduced with permission [53]. Copyright 2022, Elsevier.

The Ti_3_C_2_‐Cu/Co hybrids obtained through LAMS etching, confirm the functionality derived from interfacial interactions between metallic species from the Lewis acid and MS‐MXene [52]. These metallic species were stably retained in H_2_SO_4_, distinct from the residual salt and aggregated metal particles, which act in synergy with the MXene matrix and delivered exceptional electrochemical energy storage capability. Considering the excellent electrochemical activities of Cu/Co compounds, they chose CuCl_2_ and CoCl_2_ as the etchants to obtain Ti_3_C_2_‐Cu and Ti_3_C_2_‐Co (Figure 5a). Meanwhile, the bimetallic hybrids of Ti_3_C_2_‐Cu_1_Co_2_, Ti_3_C_2_‐Cu_1_Co_4_, and Ti_3_C_2_‐Cu_1_Co_8_ were obtained by the two salts mixture with the Cu/Co ratio of 1:2, 1:4, and 1:8, respectively. Residual salt can be removed by deionized water washing and the more stable metal particles are retained in order on the surface of the lamellar structure of Ti_3_C_2_T_x_ (Figure 5b). The interplanar distance of 2.09 Å ascribed to (111) crystal plane of Cu or Co, together with the (002) facet of Ti_3_C_2_ and polycrystalline characteristics of Ti_3_C_2_ with Cu/Co in the selected area electron diffraction (SAED) patterns (Figure 5c), manifests the successful synthesis of Ti_3_C_2_‐Cu/Co composite. The uniform distribution of the five elements (Figure 5d) verifies the ordered Cu/Co metal atoms arrangement. XRD patterns (Figure 5e) further proves the coexistence of the metallic Cu, Co atoms and the MS‐Ti_3_C_2_.

The Sn‐nanoconfined Ti_3_C_2_T_x_ MS‐MXene composites can also be synthesized in one step, simply using SnCl_2_ as Lewis acid salt, followed by deionized water washing to remove unnecessary residual salts, freeze‐drying treatment to exfoliate Ti_3_C_2_T_x_/Sn composites (Figure 5f). The avoidance of the traditional ex situ mixing method during synthesis of MXene‐based composites enables good structural stability of metal/MS‐MXene and the 2D layer‐confined effect also hinders the coarsening of metal Sn during high temperature synthesis process and the electrochemical cycles [53]. The Ti_3_C_2_T_x_/Sn composite also achieved a remarkable enhancement for Li‐storage capability during long‐term cycle. The XRD patterns (Figure 5g) exhibit the successful etching of Ti_3_AlC_2_ and the well‐preserved Sn metal. Notably, the SnCl_2_ alone cannot successfully etch Ti_3_AlC_2_ thermodynamically, but with a eutectic NaCl/KCl salt mixture as the reaction medium, the etching process can be done normally [53]. This suggests that the eutectic salt mixture environment may enhance the etching ability of a specific molten salt, lowering the overall energy consumption. The morphology of sandwich‐like nanoconfined Sn/Ti_3_C_2_T_x_ MXene (Figure 5h) is verified by smaller Sn nanoparticles anchored at the edge of Ti_3_C_2_T_x_ MS‐MXene. The interlayer spacing of around 1.15 nm can be observed by HRTEM image (Figure 5i), which is larger than that of previously reported HF‐MXene owing to the presence of metallic nanoparticles. The distribution of Sn nanoparticles (Figure 5j) on the nanosheet basal plane and edge areas, and the uniform distribution of Ti, and Cl‐termination can be verified. The effect of etching temperature must also be considered when selecting a suitable etchant for a specific LAMS etching process. The temperature‐dependent results (Figure 5k,l) from 450°C to 700°C, indicate the complete phase transformation to Ti_3_C_2_T_x_ at 600°C by the (002) peak shift from 9.51° to 8.16°, together with the complete disappearance of (104) peak, and the decreasing Al signals with increasing reaction temperature in Al 2p spectra. The pristine architecture of Sn‐nanoconfined Ti_3_C_2_T_x_ composite was also retained after long‐term cycling tests (Figure 5m). The retained Sn metal particles prove the stable and robust structure of Sn‐nanoconfined Ti_3_C_2_T_x_.

**FIGURE 6:**
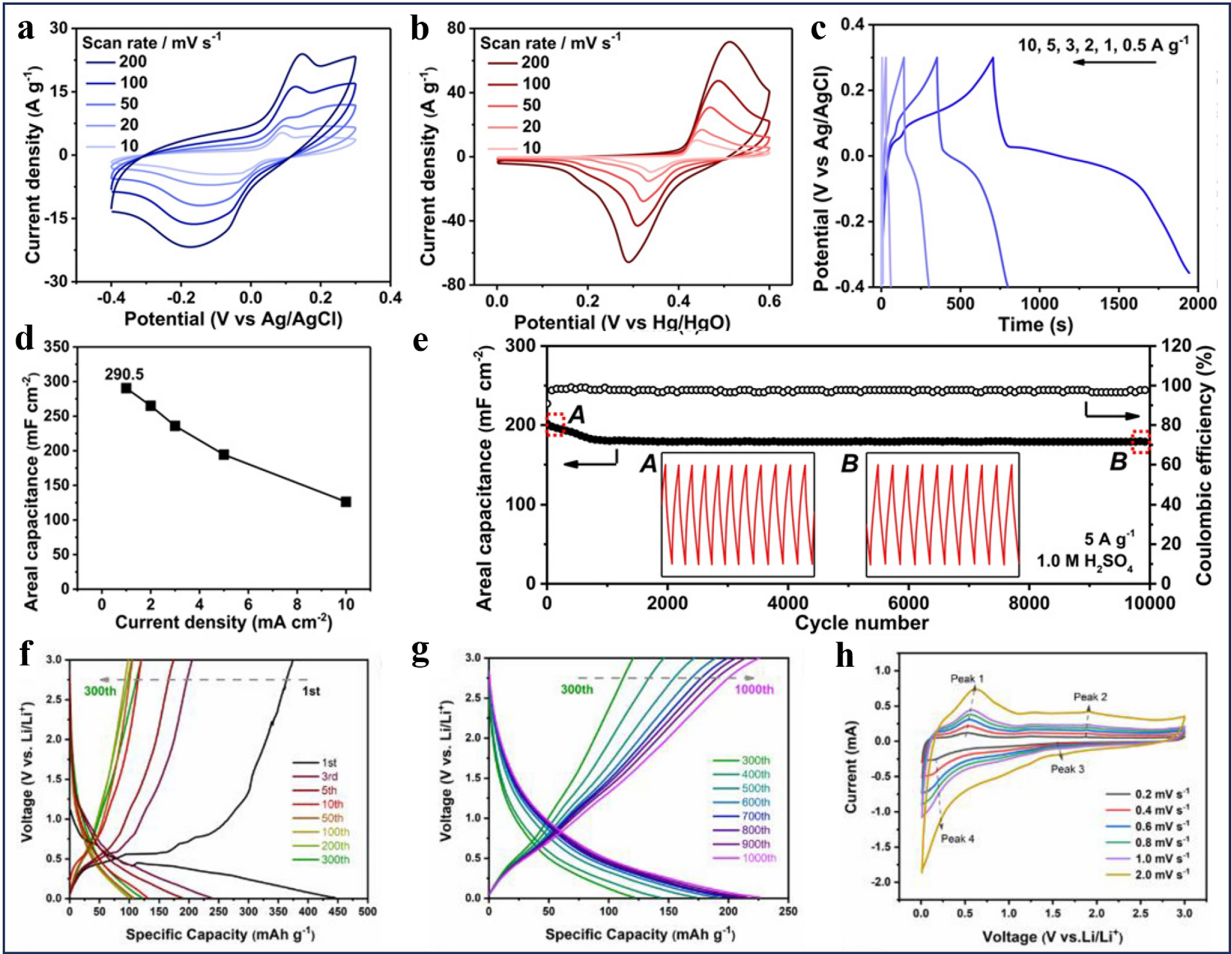
CV curves of Ti_3_C_2_‐Cu at different scan rates, in a) H_2_SO_4_ and b) KOH electrolytes at different scan rates. c) GCD curves of Ti_3_C_2_‐Cu in H_2_SO_4_. d) Areal capacitance as a function of current density for Ti_3_C_2_‐Cu//Ti_3_C_2_‐Cu. e) Long‐term cycling of Ti_3_C_2_‐Cu//Ti_3_C_2_‐Cu over 10 000 GCD cycles at a current density of 5 A g^−1^. Inset: GCD curves extracted from regions A and B [52]. Reproduced with permission [52]. Copyright 2021, John Wiley and Sons. f) GCD curves of Ti_3_C_2_T_x_/Sn composites during the initial cycling stages at 200 mA g^−1^. g) GCD curves of Ti_3_C_2_T_x_/Sn composites during prolonged cycling at 200 mA g^−1^. h) CV profiles of Ti_3_C_2_T_x_/Sn composites at various scan rates from 0.2 mV s^−1^ to 2 mV s^−1^ [53]. Reproduced with permission [53]. Copyright 2022, Elsevier.

When the as‐obtained Ti_3_C_2_‐Cu was applied in a symmetric supercapacitor, which was termed Ti_3_C_2_‐Cu//Ti_3_C_2_‐Cu, it exhibited a superior capacitance of 885.0 F g^−1^ at 0.5 A g^−1^ in 1.0 M H_2_SO_4_, which was ascribed to the pseudo‐capacitive contribution of Cu. The CV curves within the potential window of −0.4 to 0.3 V at scan rates of 10–200 mV s^−1^ in H_2_SO_4_ (Figure 6a) demonstrate the fast, diffusion‐independent redox behavior of surficial Cu atoms. In alkaline electrolyte, the CV curves (Figure 6b) display well‐separated oxidation and reduction peaks, and characteristic of a battery‐type redox process. The peak currents increase with scan rates rising (10–200 mV s^−^^1^), accompanied by slight potential shifts, which indicates a diffusion‐controlled charge storage behavior in contrast to the nearly capacitive response in acidic electrolyte. The GCD curves of Ti_3_C_2_‐Cu symmetric supercapacitors in both electrolytes (Figure 6c) are consistent with the CV results, exhibiting sloping discharge profiles with distinct plateaus. A capacitance of 290.5 mF cm^−2^ at 1 mA cm^−2^ can be retained at 89% even after 10 000 GCD cycles (Figure 6d,e). The surface terminated‐oxygen groups are usually deemed to connect metallic clusters with MS‐MXene, which provides the enhanced interaction and improved charge transfer kinetics [9]. The obvious increase in specific charge capacity of 104.1, 120.6, 146.2, 171.5, 189.2, 199.3, 206.2, 214.5, 225.6 mAh g^−1^ at the 200th, 300th, 400th, 500th, 600th, 700th, 800th, 900th, 1000th cycles can be seen in the GCD curves (Figure 6f,g). The kinetics of Ti_3_C_2_T_x_/Sn composite anode toward Li‐storage are depicted by CV curves (Figure 6h) at various scan rates from 0.2 to 2 mV s^−1^.

Lewis acid‐derived metal species should not be regarded merely as residual components, but as chemically active surface states that influence subsequent reactivity and functional behavior. From a data‐oriented perspective, surface metal incorporation should therefore be treated beyond termination chemistry alone. Relevant variables include MXene matrix composition, termination chemistry, metal identity, confinement state, and interfacial coordination, all of which jointly influence electronic structure, charge‐transfer behavior, and functional response. While metal content, interlayer spacing, and spatial distribution can be experimentally evaluated, more localized features such as bonding strength and confinement geometry often require spectroscopy‐assisted interpretation and DFT‐supported models. Such considerations enable retained metal species to be consistently incorporated into future data‐driven analysis of LAMS‐derived MXenes.

### 3.3 Surface Directed Heterostructure Engineering

Beyond intrinsic MS‐MXene properties and LAMS‐derived surface metal incorporation, functional modification can be further integrated into the synthetic pathway. In this context, LAMS chemistry is not limited to generating halogen‐terminated MXenes or metal‐containing surfaces. Instead, it can provide a reactive environment in which surface metal regulation and interface formation occur in a coupled manner. Such pathways are particularly valuable because functional phases and modified interfaces can be generated as an integrated part of the LAMS conversion process. Compared with termination engineering or surface metal incorporation, heterostructure engineering represents a higher level of surface‐chemistry complexity. In these systems, the final functionality is governed not only by the MXene matrix, termination chemistry, or retained metal state, but also by how the interface is constructed and coupled with the LAMS‐derived surface. Consequently, interfacial bonding, spatial distribution, and structural integration become critical factors governing the final physicochemical behavior. The following examples illustrate how LAMS‐derived surfaces can direct heterointerface formation through both in situ conversion and ex situ pathways.

**FIGURE 7:**
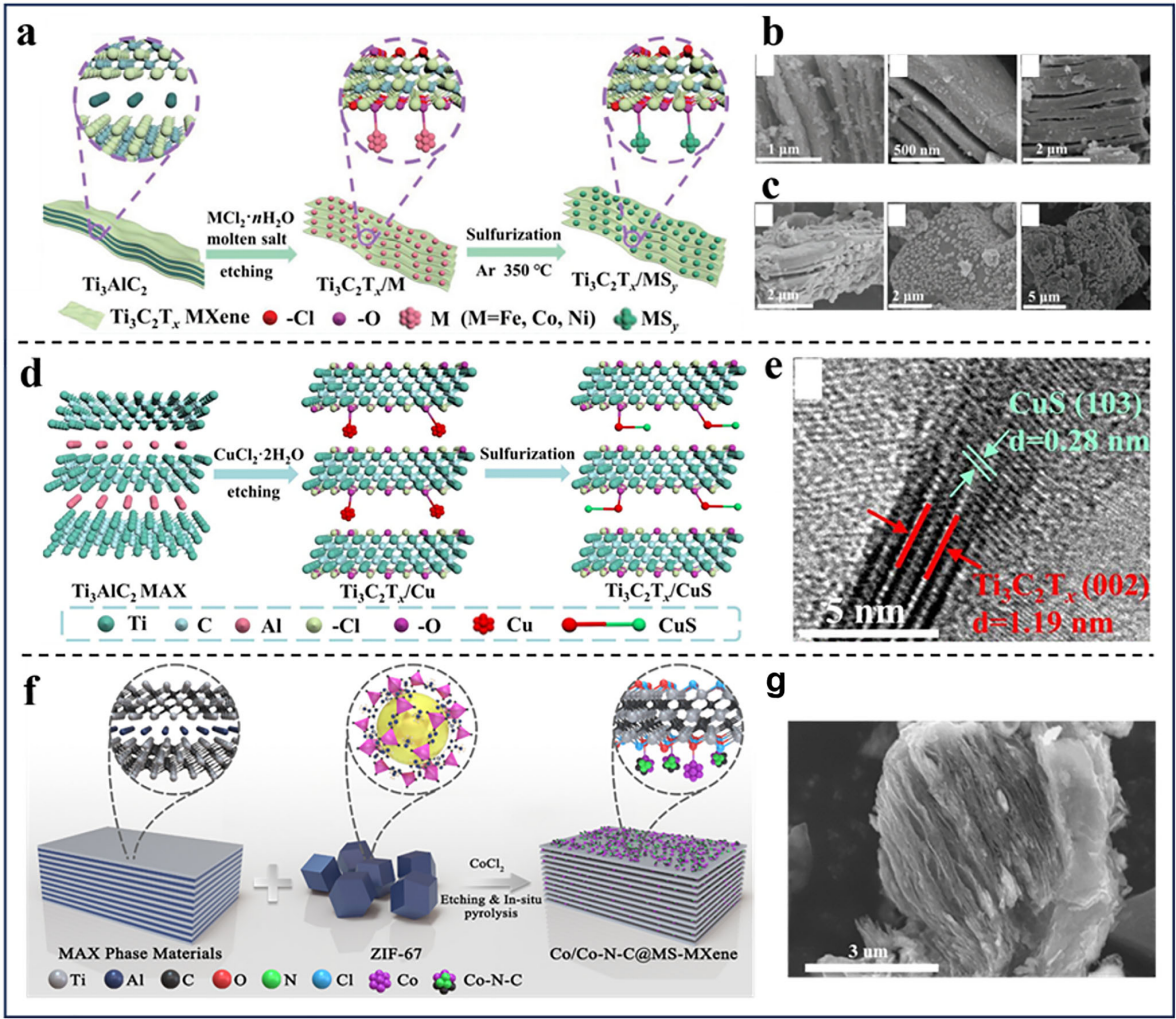
a) Schematic illustration of the synthesis of of Ti_3_C_2_T_x_/MS_y_ heterostructures. b) SEM images of Ti_3_C_2_T_x_/Fe, Ti_3_C_2_T_x_/Co, Ti_3_C_2_T_x_/Ni, and c) Ti_3_C_2_T_x_/FeS_2_, Ti_3_C_2_T_x_/CoS_y_, Ti_3_C_2_T_x_/NiS_y_, from left to right [55]. Reproduced with permission [55]. Copyright 2022, John Wiley and Sons. d) Schematic illustration of the synthesis of the Ti_3_C_2_T_x_/CuS hybrids. e) HRTEM image of Ti_3_C_2_T_x_/CuS [56]. Reproduced with permission [56]. Copyright 2022, Royal Society of Chemistry. f) In situ synthesis of Co/Co‐N‐C@MS‐MXene. g) SEM image of Co/Co‐N‐C@MS‐MXene [57]. Reproduced with permission [57]. Copy right 2025, American Chemical Society.

Early examples of LAMS‐coupled in situ heterostructure formation involved CoCl_2_‐derived MX‐Co, where surface‐anchored Co species were directly converted into MX‐CoS_2_ or N‐MX‐ CoS_2_ through sulfidation using sulfur or thiourea [71, 72]. The synthetic protocol (Figure 7a) of Ti_3_C_2_T_x_/MS_y_ was reported by Han's group [55]. MCl_2_·nH_2_O was used as LAMS etchants, followed by thiourea‐assisted sulfidation to convert the surface‐anchored metal species into corresponding metal sulfides. The stepwise formation of Ti_3_C_2_T_x_/Fe, Ti_3_C_2_T_x_/Co, Ti_3_C_2_T_x_/Ni (Figure 7b), followed by their transformation into Ti_3_C_2_T_x_/FeS_2_, Ti_3_C_2_T_x_/CoS_y_, Ti_3_C_2_T_x_/NiS_y_ (Figure 7c), shows the morphological changes associated with the conversion of surface‐anchored transition‐metal atoms into their metallic sulfide phases.

The MXene/CuS hybrids were synthesized via the MS^3^ strategy, using CuCl_2_·2H_2_O as the Lewis acid etchant (Figure 7d) [56]. The heterointerface between the Ti_3_C_2_T_x_ (002) and CuS (103) crystal planes (Figure 7e) confirms the successful synthesis of Ti_3_C_2_T_x_/CuS. Moreover, the in‐situ pyrolysis of ZIF‐67 could also be coupled into the LAMS etching process [57] (Figure 7f), forming MOF‐derived carbon decorated Co/Co‐N‐C@MS‐MXene (Figure 7g).

**FIGURE 8:**
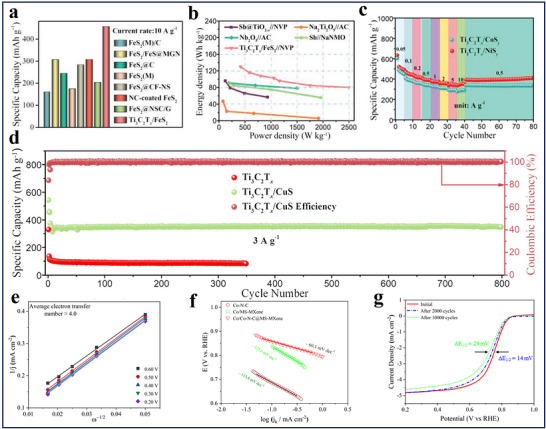
a) Rate performance comparison at 10 A g^−1^ between reported FeS_2_‐based SIBs anodes and Ti_3_C_2_T_x_/FeS_2_ electrode. b) Ragone plots comparison of various full‐cell devices. c) Rate capability of Ti_3_C_2_T_x_/CoS_y_ and Ti_3_C_2_T_x_/NiS_y_ electrodes. Reproduced with permission [55]. Copyright 2022, John Wiley and Sons. d) Long‐term cycling performance and coulombic efficiency of the Ti_3_C_2_T_x_ and Ti_3_C_2_T_x_/CuS electrodes at 3 A g^−1^. Reproduced with permission [56]. Copyright 2022, Royal Society of Chemistry. e) K‐L plots of ORR on the Co/Co‐N‐C@MS‐MXene. f) Tafel slope comparison between Co‐N‐C, Co/MS‐MXene, and Co/Co‐N‐C@MS‐MXene. g) Stability test of Co/Co‐N‐C@MS‐MXene heterostructure [57]. Reproduced with permission [57]. Copyright 2025, American Chemical Society.

Such LAMS‐derived heterostructures have demonstrated promising electrochemical functionalities in sodium‐ion batteries and electrocatalysis. For example, Ti_3_C_2_T_x_/FeS_2_, Ti_3_C_2_T_x_/CoS_y_ and Ti_3_C_2_T_x_/NiS_y_ generated through in situ sulfidation exhibited improved performance and long‐term stability as anodes for sodium‐ion batteries (SIBs), demonstrating the effectiveness of converting LAMS‐retained surface metal species into functional sulfide interfaces (Figure 8a–c) [55]. Similarly, the Ti_3_C_2_T_x_/CuS heterostructure showed enhanced stability associated with the formation of Ti─O─Cu interfacial bonding, emphasizing the critical role of interface chemistry (Figure 8d) [56]. Beyond sulfide heterostructures, the MOFs‐derived Co/Co‐N‐C@MS‐MXene delivered an efficient oxygen reduction reaction (ORR) activity with a four‐electron reaction pathway, where the strong electronic coupling between MOF‐derived carbon and LAMS‐derived MXene facilitated interfacial charge transfer and catalytic regulation (Figure 8e–g) [57]. These in situ examples demonstrate that LAMS‐derived MXenes can integrate etching, metal retention, and interface‐forming conversion within a chemically continuous pathway. Rather than functioning merely as etching residues, retained metal species act as built‐in precursors that participate directly in heterointerface formation. Consequently, the resulting interface is governed not only by final composition but also by the sequence of chemical transformation and conversion history.

Ex situ heterostructure engineering follows a different logic. In this case, the LAMS‐derived MXene surface has already been established prior to secondary treatment, and the key question becomes how the predefined surface state directs subsequent deposition, assembly, or interfacial coupling. The LAMS‐defined surface can serve as a chemically selective platform that guides nucleation, regulates interfacial matching, and promotes structural assembly. For example, the incorporated metal species can directly regulate additional metallic deposition by defining chemically specific nucleation interfaces [73]. In Zn‐modified Ti_3_C_2_Cl_2_ derived from ZnCl_2_, the uniformly distributed residual Zn sites on the MXene surface act as lithophilic nucleation centers, lowering the nucleation barrier of Li and homogenizing the local ion flux and electric field during plating [74]. The key role of the MXene here is therefore not merely structural support, but fine‐tuned platform for Li deposition that suppresses the dendrite growth [58]. A related strategy, halogen‐terminated Ti_3_C_2_T_x_, obtained from Cu halide molten salt etching (CuCl_2_, CuBr_2_, CuI), regulates Zn deposition through termination‐dependent interfacial matching [32]. Here, the role of Cl, Br, and I terminations is not merely to alter surface adsorption, but to reshape the lattice compatibility and interfacial deposition energetics of Zn, among which Cl termination provides the most favorable deposition microenvironment [75]. The surface chemistry‐dependent Zn deposition behavior further demonstrates the critical role of halogen termination in regulating interfacial compatibility and deposition kinetics.

Another category of ex situ heterostructure engineering involves structural transformation and interfacial assembly. For example, a sodium alginate‐assisted hydrothermal process was proved to convert Ti_3_C_2_Cl_2_ nanosheets into nanodots [60], while the Cl‐terminated surface provides a favorable platform for the subsequent transformation and interfacial assembly for layered double hydroxide (LDH). The resulting QD‐Ti_3_C_2_Cl_2_@NiAl‐LDHs heterostructure further demonstrates that halogen termination relaxes the surface chemistry constraints imposed by strong Ti─O bonds, providing a more reactive and chemically accessible platform for subsequent functionalization [76].

From a data‐driven perspective, both in situ and ex situ heterostructure engineering should be treated as heterointerface‐level problems rather than composition‐ or termination‐only problems. Relevant descriptors should capture both processing history and interface characteristics. Directly recordable variables include modification route, precursor or reagent source, reaction temperature, reaction time, atmosphere, loading level, and post‐modification treatment. Modified‐state composition, spatial distribution, and interface morphology can be partially resolved through diffraction, spectroscopy, and microscopy. More features, including local chemical environment, bonding configuration, and pathway‐dependent heterogeneity, remain challenging to quantify directly and may require combined spectroscopy, microscopy, and DFT‐supported interface models. Such distinction is important because identical nominal MXene compositions may produce substantially different functional responses depending on how the heterointerface is generated, highlighting the necessity of incorporating process‐dependent descriptors into future data‐driven models.

### 3.4 Post‐Synthetic Surface Modification

Post‐synthetic regulation can further tailor the surface chemistry, interlayer environment, and accessibility of LAMS‐derived MXenes through delamination, termination transformation, and structural regulation [77, 78]. Unlike heterostructure‐forming pathways discussed in Section 3.3, these treatments generally preserve MXene identity while modifying its local chemical or structural state.

Delamination represents one of the most effective post‐treatment strategies [79]. Bulky intercalants or solvated ions can weaken interlayer interactions and improve wettability, colloidal stability, and electrochemical accessibility [65, 66]. For example, tetrabutylammonium assisted or electrochemical swelling‐delamination treatments preserved the original halogen‐defined surface chemistry while expanding layered structures and enhancing active‐surface utilization. These results indicate that delamination should not be viewed merely as physical exfoliation, but rather as a coupled regulation of interlayer chemistry and transport behavior.

Surface chemistry can be further tuned through aqueous or molten‐salt treatments. Exposure of Ti_3_C_2_Cl_2_ to H_2_O, H_2_SO_4_, NaOH, or APS altered termination populations without fully disrupting the layered morphology, resulting in markedly different electrochemical performance [69]. Beyond aqueous chemistry, halogen‐terminated MXenes also undergo direct molten‐salt transformation owing to the relatively weak M─Cl and M─Br bonding, enabling the formation of diverse surface states including O‐, S‐, Se‐, and Te‐containing terminations [30]. These studies establish LAMS‐derived terminations as chemically transformable rather than static surface species.

In addition to termination regulation, post‐treatment can influence morphology and interfacial properties. Etching‐coupled porosity engineering generated mesoporous Ti_3_C_2_Cl_2_ with enhanced surface area and ion‐storage capability [68], while Ti_3_C_2_Cl_2_ incorporated into CsPbBr_3_ films relieved lattice strain and improved grain growth through termination‐mediated interfacial interactions [70]. Such examples demonstrate that the chemical functionality of LAMS‐derived surfaces can extend beyond MXene optimization itself and participate in broader materials regulation.

From a data‐oriented perspective, post‐synthetic modification should be represented as a dynamic surface‐evolution process rather than an isolated treatment step. Aqueous and molten‐salt treatments define termination‐evolution pathways through variables such as treatment chemistry, reaction environment, and final surface state, whereas delamination introduces descriptors related to interlayer accessibility, colloidal behavior, and film formation. Consequently, the functionality of LAMS‐derived MXenes depends on both the initial surface state established during etching and the dynamic surface evolution introduced during post‐treatment. While parameters such as termination population, interlayer spacing, conductivity, and colloidal or film structure can be experimentally evaluated, more dynamic processes including termination exchange, partial oxidation, and surface reconstruction often require spectroscopy‐ and calculation‐assisted interpretation. As summarized in Table 3, these variables exhibit different levels of measurability, reliability, and structural complexity. Such descriptor heterogeneity highlights a key challenge for LAMS‐derived MXenes, where synthesis history, surface evolution, and interfacial chemistry must be represented in a form suitable for predictive analysis.

**TABLE 3:** Data reliability and modeling readiness of representative LAMS‐derived MXene variables.

Data reliability level	Representative variables	Main evidence or source	ML readiness
Directly measurable	Precursor type, Lewis acid salt, salt ratio, etching temperature/time, atmosphere, interlayer spacing, yield, residual metal content	Synthesis record, XRD, elemental analysis, microscopy	High
Measurable but fitting‐dependent	Termination coverage, oxidation state, metal speciation, surface O/halogen ratio	XPS fitting, XAS, elemental balance, DFT‐assisted interpretation	Medium
Indirectly inferred	Local coordination environment, metal–matrix bonding, confinement state, interlayer ion distribution, surface charge redistribution	XAS, STEM mapping, DFT models, spectroscopy‐informed analysis	Medium to low
Poorly standardized	Washing history, delamination condition, electrochemical protocol, mass loading, normalization method	Reported experimental procedures	Low unless metadata are standardized
Currently unsuitable for direct ML labels	Transient A‐site extraction intermediates, local Lewis acidity in molten salts, exact termination ordering, dynamic surface evolution, complex multi‐component interface states	Indirect calculation or incomplete literature evidence	Unsuitable without controlled datasets or physics‐based proxies

## 4 Data‐Driven Discovery of Electrochemical Materials Based on MXenes

Such rich tunability of MXene surface chemistry creates a high‐dimensional, strongly coupled design space, rendering conventional trial‐and‐error approaches inefficient and motivating the use of data‐driven strategies [80]. Machine learning therefore plays a central role in data‐driven MXene research, both as a surrogate model for rapid high‐throughput screening and as an active learning tool for iterative materials discovery [81]. For LAMS‐derived MXenes, effective modeling relies on translating synthesis‐dependent chemistry into physically meaningful descriptors. As summarized in Table 4, the surface‐regulation routes discussed in Section 3 collectively define descriptor spaces involving surface terminations, retained metal states, heterointerfaces, and post‐synthetic treatments, emphasizing the importance of surface and interfacial information beyond nominal composition alone.

**TABLE 4:** Descriptor categories, characterization accessibility, and property relevance of representative LAMS‐derived MXene variables.

Surface regulation route	Descriptor type	Representative variables	Characterization source	Data availability	Modeling difficulty	Relevant property
LAMS synthesis parameters	Controllable synthesis descriptors	MAX precursor, Lewis acid salt, eutectic salt, salt ratio, temperature, time, atmosphere	Experimental procedure	High for reported conditions; lower when washing history or yield is omitted	Low to medium	Phase conversion, yield, termination formation
Halogen terminations	Categorical surface descriptor	Cl, Br, I, multi‐halogen design	XPS, elemental analysis, EDS, DFT	Relatively high	Low to medium	Work function, ion adsorption, redox behavior
Retained metal species	Metal state descriptor	Metal content, phase, oxidation state, spatial distribution	ICP, XPS, XAS, STEM‐based elemental mapping	Medium; speciation‐dependent	Medium to high	Redox activity, metal nucleation, catalytic sites
Complex heterostructures	High‐dimensional heterointerface descriptor	Secondary phase identity, active‐site type, loading, spatial distribution, interface continuity	Combined XRD, XPS/XAS, microscopy, and electrochemical characterization	Low to medium; cross‐study comparability is limited	High	Catalytic selectivity, capacity, long‐term durability
Post‐synthetic treatments	Processing and surface‐evolution descriptor	Reagent, pH, treatment time, intercalant, delamination route	Experimental procedure, XRD, microscopy, conductivity measurement	Medium; time‐dependent	Medium to high	Colloidal stability, film conductivity, active surface utilization

High‐throughput DFT and machine learning can then identify synthesis and processing conditions most likely to generate targeted surface states, while interpretable models help clarify which descriptors govern adsorption, charge redistribution, ion transport, and interfacial stability. Instead of screening final compositions alone, such approaches enable closed‐loop optimization linking precursor design, LAMS chemistry, post‐treatment, and functional response. Representative studies are further summarized in Table 5 according to their roles in connecting synthesis‐dependent chemistry with predictive materials design.

**TABLE 5:** Task‐oriented summary of data‐driven strategies for MXene discovery.

Task	Target	Representation	Data availability	Model framework	Modeling difficulty	Methodological insight	Refs.
High‐throughput screening	HER activity	Elemental, geometric, site‐specific, DFT‐derived descriptors	Medium to high	RF, GB, AdaBoost, XGBoost, ANN, multisite models	Medium	Extend DFT screening to large candidate spaces; Useful for exploring broad composition‐termination spaces	[82]
Stability‐activity co‐screening	Δ*G*_H*_, thermal stability	Elemental, geometric, and DFT‐derived descriptors	Medium to high	AdaBoost, ANN, phonon/AIMD‐assisted validation	High	Constraints beyond activity; Important for chemically complex and synthesis‐sensitive system	[83, 84]
Active‐learning stability discovery	*E*_hull_	Composition/structure descriptors, DFT updates	Medium to high	GP, expected improvement	Medium	Minimize DFT cost in large stability spaces; Suitable for compatibility mapping	[85]
Synthesizability prediction	Synthesizability	Elemental, DFT‐derived structural, thermodynamic, electronic descriptors	Medium to high	PU learning	High	Connect theoretical candidates to experimental feasibility; Highly relevant to synthesis‐informed design	[86]
Configuration‐sensitive property modeling	Conductivity, photoconductivity	Atomic configurations, disorder‐informed structural representations	Low to medium	Equivariant GNN, MC sampling	High	Property prediction in disordered configurational spaces; Useful for mixed terminations and interlayer complexity	[87]
Surrogate electronic‐property prediction	Band gap	Atomic and structural descriptors; limited GW reference data	Low to medium	Classifier, GPR, MXgap	Low	Scalable prediction; Useful for functionalized semiconducting MXene discovery	[88, 89]
Interpretable descriptor analysis	Work function, activity‐related descriptors	Elemental/electronic descriptors	Low to medium	RF, SISSO	Low	Extract descriptor‐property relations; Useful for linking surface chemistry to functionality	[90]
Spectroscopic interpretation	Raman response under disorder and mixed terminations	ML force field, structural dynamics	Medium to high	MLFF, DFT/MD	High	Connect experimental signatures to microscopic origins; Useful for complex surface characterization	[91]
Device optimization	Capacitance, cycling stability, retention	Literature‐derived experimental variables	Low to medium	CART, RF	Low	Extract optimization rules from heterogeneous experimental data; Processing‐dependent optimization	[92]
Closed‐loop inverse design	Electrical and mechanical properties of conductive MXene aerogels	Fabrication parameters; formulation variables; mechanical and conductivity for output	Low to medium	RF, GPR, ANN; SHAP, FE; BO, qEHVI; MTGPR	High	Programmable property optimization and inverse design; Synthesis‐informed closed‐loop optimization	[93]

Most existing data‐driven MXene studies focus on candidate screening and property prediction using descriptors derived from composition, surface terminations, adsorption characteristics, and DFT‐based electronic features [94]. Increasingly, however, emphasis is shifting toward chemically interpretable and experimentally accessible descriptor frameworks [95]. For LAMS‐derived MXene, descriptor maturity follows a hierarchy of chemical complexity. Termination‐related problems are currently the most suitable for high‐throughput and interpretable modeling, whereas surface metal incorporation and interface engineering introduce increasingly coupled and less standardized variables. Consequently, data‐driven discovery remains most mature for intrinsic MXene surface chemistry, while interface‐engineered LAMS systems represent a more challenging but important frontier.

### 4.1 ML‐Assisted High‐Throughput Screening

ML‐assisted high‐throughput screening has become a representative strategy for data‐driven MXene discovery, particularly for HER, where surface terminations, dopants, and local adsorption environments generate a chemical space too large for exhaustive first‐principles exploration alone [96, 97, 98]. ML models trained on DFT‐derived descriptors enable rapid prediction and extension toward much larger candidate spaces.

**FIGURE 9:**
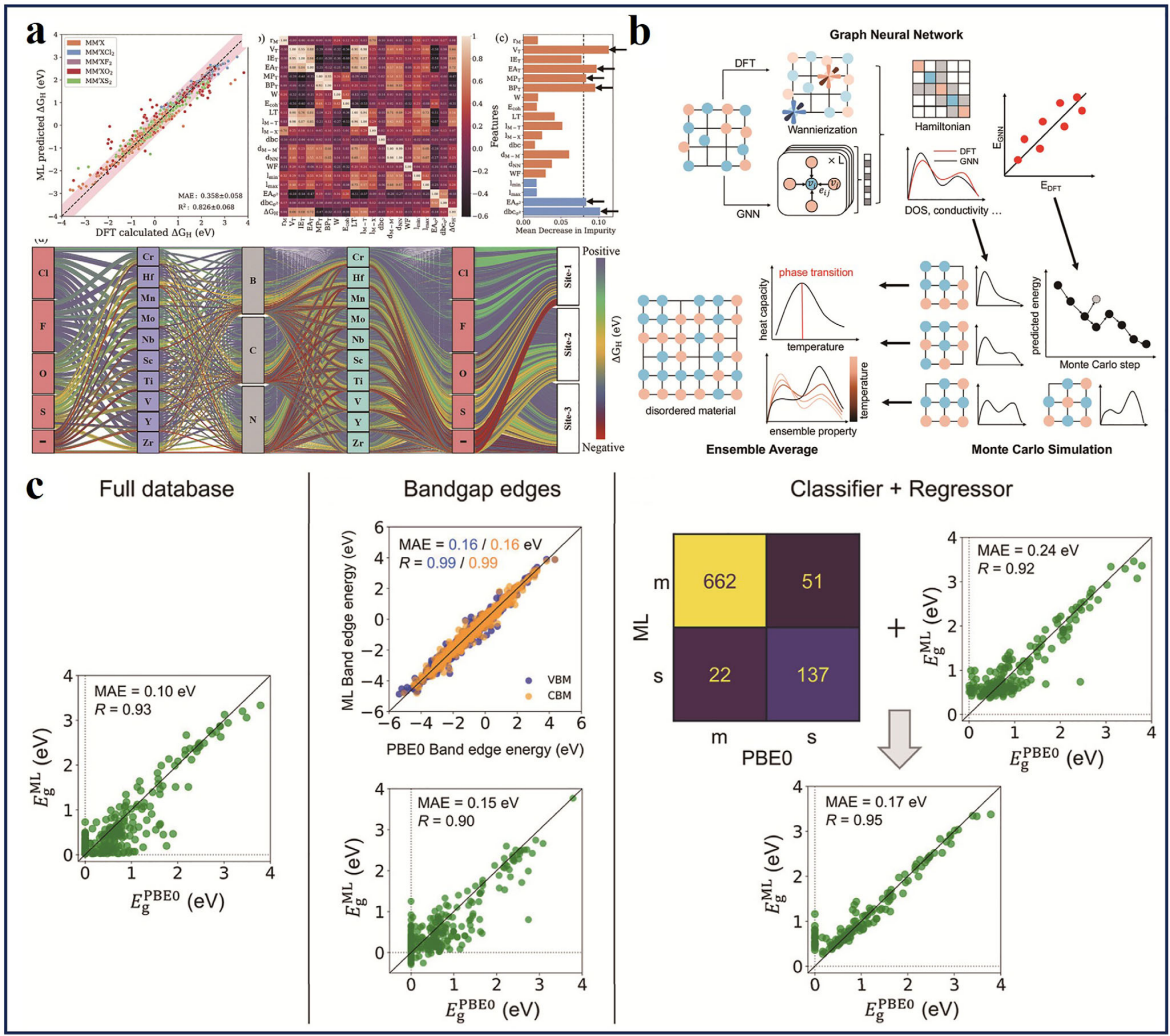
Representative applications of ML in MXene research. a) ML‐assisted property prediction and composition‐property relationship analysis, including impurity formation energy prediction and feature‐importance analysis [101]. Reproduced with permission [101]. Copyright 2023, Royal Society of Chemistry. b) Integration of graph neural networks, DFT calculations, and Monte Carlo (MC) simulations for predicting the properties of disordered systems and analyzing collective behaviors such as phase transitions [87]. Reproduced with permission [87]. Copyright 2025, American Chemical Society. c) ML for predicting band gaps and band edge positions, with improved classification of metallic/semiconducting states and enhanced bandgap prediction through a combined classifier and regressor strategy [89]. Reproduced with permission [89]. Copyright 2025, American Chemical Society.

For HER catalysis, multisite adsorption prediction represents an important screening strategy because MXene surfaces often exhibit chemically distinct adsorption configurations. Based on high‐throughput DFT datasets of TM‐doped Ti_3_CNO_2_ and Zr_2_HfCNO_2_ systems, multisite ML models were developed to predict hydrogen adsorption free energy (Δ*G*_H_*) across multiple adsorption sites. By integrating DFT‐derived, elemental, and site‐related descriptors, Gradient Boosting (GB) [99] and Random Forest (RF) [100] models achieved effective prediction while maintaining strong performance even after removal of computationally expensive features. Subsequent screening identified Nb, Sc, Rh, W, Ti, and V as promising dopants for HER catalysis [82]. For the much larger configurational space of MM′XT_2_‐type MXenes, similar high‐throughput DFT‐ML workflows further demonstrated the ability of supervised learning models to efficiently screen uncalculated candidates for HER activity (Figure 9a) [101].

Beyond activity prediction alone, stability‐activity co‐screening has emerged as a more practically relevant strategy for MXene catalyst discovery. One route employs stability‐constrained dataset construction, where O‐terminated ordered binary alloy MXenes screened by high‐throughput DFT were used to establish descriptor‐activity relationships [83]. AdaBoost analysis further highlighted key descriptors associated with Δ*G*_H_*, including local bonding environments and electronic characteristics [102]. A complementary strategy applied descriptor sets composed of readily accessible elemental properties to single‐atom and termination‐engineered Ti_3_C_2_T_2_ systems [84]. Among the tested models, artificial neural networks (ANN) [103] showed strong predictive capability for simultaneous HER activity and thermal stability assessment, while phonon calculations and ab initio molecular dynamics (AIMD) [104] further validated the stability of screened candidates.

Similar descriptor‐guided workflows have also been extended to MXene‐based energy‐storage exploration [105, 106], demonstrating how ML‐assisted workflows can efficiently identify activity‐ and stability‐related descriptors for MXene electrocatalysts. Similar descriptor‐guided strategies can be readily extended to other electrocatalytic and electrochemical energy‐storage systems [107, 108].

### 4.2 ML‐Assisted Evaluation of Stability and Synthesizability

ML has also been used to reduce the MXene search space by targeting stability and synthesizability, two criteria that are directly linked to whether data‐driven candidates can move beyond theoretical prediction [109]. Thermodynamic stability, in particular, has become an important focus, and unlike the supervised screening workflows discussed above, one recent study adopted an active learning strategy to explore the stability of MXenes over a broad chemical space [85]. Specifically, the energy above the convex hull (*E*_hull_) was used as the thermodynamic stability criterion, and a Gaussian process (GP) [110] model was trained to predict *E*_hull_. Coupled with an expected improvement strategy, the model iteratively selected the most promising candidate structures for further DFT calculations. Ultimately, among 23 857 MXene candidates, 126 thermodynamically stable materials were identified using only 480 additional DFT calculations, highlighting the efficiency of active learning for large‐scale stability screening.

Data‐driven approaches have also provided a new perspective for evaluating the feasibility of translating theoretically designed candidates into experimentally realizable materials. In this context, an emerging ML method, positive‐unlabeled learning (PU‐learning) [111], has been applied to the study of the synthesizability of MXenes and their precursor MAX phases [86]. Specifically, experimentally synthesized materials were treated as positive samples, whereas materials that had not yet been synthesized were regarded as unlabeled samples. By combining elemental information with structural, thermodynamic, and electronic descriptors derived from DFT calculations, the model was able to rank each candidate by its likelihood of experimental realization. The results showed that the model achieved a true positive rate (TPR) of over 90% for MAX phases and an identification positive rate (IPR) of over 75% for MXenes. Further feature analysis revealed that cohesive energy and formation energy were the two most influential descriptors governing synthesizability.

### 4.3 ML‐Assisted Property Modeling and Interpretability

Unlike screening‐oriented workflows that primarily prioritize candidates, interpretability‐focused ML seeks to clarify physically meaningful links between surface chemistry, local configuration, and target properties [112, 113]. For MXenes, such modeling becomes particularly important because surface terminations, disorder, and local chemical environments can strongly regulate electronic behavior and are often difficult to resolve through composition alone [114].

One important direction concerns electronic transport in disordered MXenes. The introduction of equivariant GNNs [115] (Figure 9b) has supported models to effectively learn the relation between atomic configurations and electronic properties from DFT/Wannier [116] derived data. The trained models were further embedded into MC [117] sampling, allowing efficient exploration of large‐scale disordered configurational spaces [87]. Using this strategy, the energy, electrical conductivity, and photoconductivity spectra of atomically disordered MXenes were predicted with mean absolute percentage errors (MAPE) of only 0.1%, 2.7%, and 3.8% on the test set, respectively. The predicted results also showed good agreement with DFT calculations. Unlike the screening studies, this framework examined how local disorder reshapes electronic response across a large configurational space.

Band gap prediction provides another representative example, especially because surface functionalization can strongly alter the electronic structure of MXenes as we mentioned before. A DFT‐integrated ML framework has been developed for the high‐throughput prediction of band gaps in functionalized MXenes [88]. In this framework, a classification model was first used to identify finite‐band‐gap systems from 23 870 candidate structures. Based on 76 GW band gap data points, several regression models, including KRR, support vector regression (SVR) [118], GP, and Bagging, were compared. The results showed that GP achieved the best predictive performance, with RMSE as low as 0.14 eV on the test set, while avoiding the need for computationally expensive electronic‐structure descriptors such as PBE band gaps. These findings indicate that ML can serve as an effective alternative to costly GW calculations [119] for rapid band gap prediction in MXenes. The packaged model MXgap further advanced this direction by translating band‐gap prediction into a deployable screening tool for MXene semiconductors and photocatalysts [89]. Built on a dataset containing 4356 MXene structures, MXgap adopted a two‐step classifier‐regressor framework, first distinguishing metallic and semiconducting systems, followed by quantitative band‐gap prediction. The best‐performing workflow (Figure 9c) achieved a classification accuracy of 92% and a mean absolute error (MAE) of 0.17 eV. Furthermore, MXgap was applied to screen 396 La‐based MXenes, leading to the identification of six candidate materials suitable for photocatalytic water splitting. In this sense, MXgap further translates band‐gap surrogate modeling into a deployable tool for rapid MXene semiconductor and photocatalyst evaluation.

Beyond conductivity and band gap, interpretable ML has also been used to quantify structure–property relationships for other key electronic descriptors. As an important quantity reflecting surface electron binding strength and surface electronic characteristics, the work function has been predicted in double‐transition‐metal MXenes using RF models trained on DFT data for 242 (M1)_2_(M_2_)X_2_O_2_ structures [90]. By further combining RF with SISSO [120] descriptor analysis, this work moved beyond prediction to reveal explicit quantitative relations between elemental features and work function, providing a more interpretable picture of how surface chemistry regulates electronic response. Similar ML‐assisted frameworks have also been extended to spectroscopic interpretation, where ML force fields and Raman‐tensor reconstruction were used to analyze termination‐ and disorder‐dependent vibrational signatures in Ti_3_C_2_T_x_ MXene [91]. Together, these studies illustrate how ML‐assisted property modeling is increasingly evolving from predictive fitting toward chemically interpretable analysis of MXene surface chemistry and local configurational effects [13].

### 4.4 Closed‐Loop and Autonomous Discovery

**FIGURE 10:**
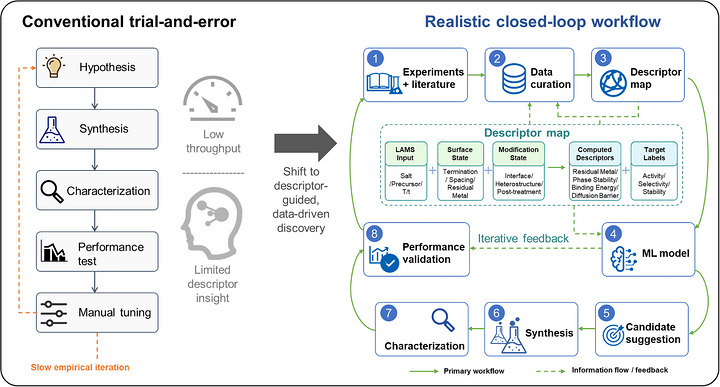
Descriptor‐guided closed‐loop workflow for LAMS‐derived MXene discovery. Comparison between conventional empirical materials discovery and a data‐driven closed‐loop workflow. The embedded descriptor map correlates LAMS synthesis parameters, surface and interlayer chemistry, descriptor space, and target performance.

Beyond candidate screening and property prediction, emerging data‐driven technologies are progressively reshaping MXene discovery toward closed‐loop and autonomous workflows [121]. In contrast to conventional trial‐and‐error synthesis, closed‐loop discovery integrates model prediction, experimental validation, and iterative feedback, enabling synthesis knowledge to be continuously updated and reused [122]. Figure 10 illustrates a descriptor‐guided workflow tailored for LAMS‐derived MXenes, where precursor selection, Lewis acid or eutectic salt design, reaction conditions, and post‐treatment variables are linked with structural and functional outputs. Rather than relying on empirical optimization alone, such workflows establish feedback between synthesis, surface states, and functional response, allowing model predictions to guide subsequent experimental design.

Despite this promise, direct implementation of closed‐loop optimization for LAMS‐derived MXenes remains challenging [123]. High‐temperature molten salts, reactor compatibility, atmosphere control, salt containment, and multi‐step post‐treatment complicate both automation and in‐line characterization. Consequently, current examples should be regarded as methodological guidance rather than demonstrations of mature autonomous LAMS etching systems. Beyond hardware and automation challenges, data quality remains another important limitation for closed‐loop MXene discovery. Key variables including synthesis conditions, surface assignment, post‐treatment history, and electrochemical testing are often reported in non‐uniform formats, limiting descriptor reliability and model transferability. Table 6 summarizes representative data‐quality barriers together with practical strategies for improving MXene dataset consistency and modeling readiness.

**TABLE 6:** Data‐quality barriers and possible improvement strategies for MXene datasets.

Data source	Useful information	Common noise or missing information	Impact on model reliability	Possible improvement strategy
Synthesis reports	Precursor, Lewis acid salt, eutectic medium, salt ratio, temperature, time, atmosphere	Incomplete salt ratio, unclear atmosphere, missing yield, incomplete post‐synthetic history	High	Minimum LAMS metadata checklist, missing‐field tagging, unit normalization, and synthesis‐condition consistency checking
XPS and surface analysis	Termination type, oxidation state, metal speciation	Inconsistent peak fitting, missing constraints, limited raw spectra	Critical	Physics‐constrained label validation, DFT‐assisted assignment plausibility scoring, confidence‐tagged records
XRD and microscopy	Phase conversion, interlayer spacing, morphology, metal distribution	Limited, statistics, local sampling bias, unclear phase purity	Moderate to high	Structure‐informed feature extraction, image‐derived descriptor mining, sampling‐bias tagging, uncertainty‐aware morphology labels
Electrochemical testing	Capacity, rate capacity, cycling stability, overpotential, yield, efficiency	Different mass loading, unnormalized protocols	Critical	Protocol normalization, metadata‐conditioned performance comparison, unit normalization
Literature mined records	Cross paper synthesis‐property relations	Missing negative results, publication bias, inconsistent terminology	High	Human‐in‐the‐loop curation, confidence tagging, source‐linked extraction

In the optimization of MXene‐based supercapacitor performance, one study extracted more than 7500 experimental data points from the published literature. Classification and regression tree [124] (CART) analysis was used to identify the key factors affecting specific capacitance, cycling stability, and capacitance retention, and RF models were subsequently developed for performance prediction [92]. In the galvanostatic charge–discharge (GCD) [125] dataset, the *R* ^2^ for specific capacitance, cycle number, and capacitance retention reached 0.87, 0.83, and 0.85, respectively. Further feature‐importance analysis revealed that factors such as working‐electrode composition, electrolyte type, substrate, and etching conditions significantly influence device performance. Based on model‐guided screening, the authors further carried out electrode design and experimental validation, demonstrating that this data‐driven strategy can not only summarize patterns hidden in the literature but also provide practical guidance for the optimization of MXene supercapacitors. This study represents a model‐assisted performance analysis based on literature‐mined MXene data. Its main value lies in showing how scattered experimental records can be converted into interpretable descriptors for performance prediction and electrode design. For LAMS‐derived MXenes, this type of analysis provides transferable methodological guidance, but reliable application still requires more consistent reporting of synthesis conditions, surface chemistry, post‐treatment history, and electrochemical testing protocols.

**FIGURE 11:**
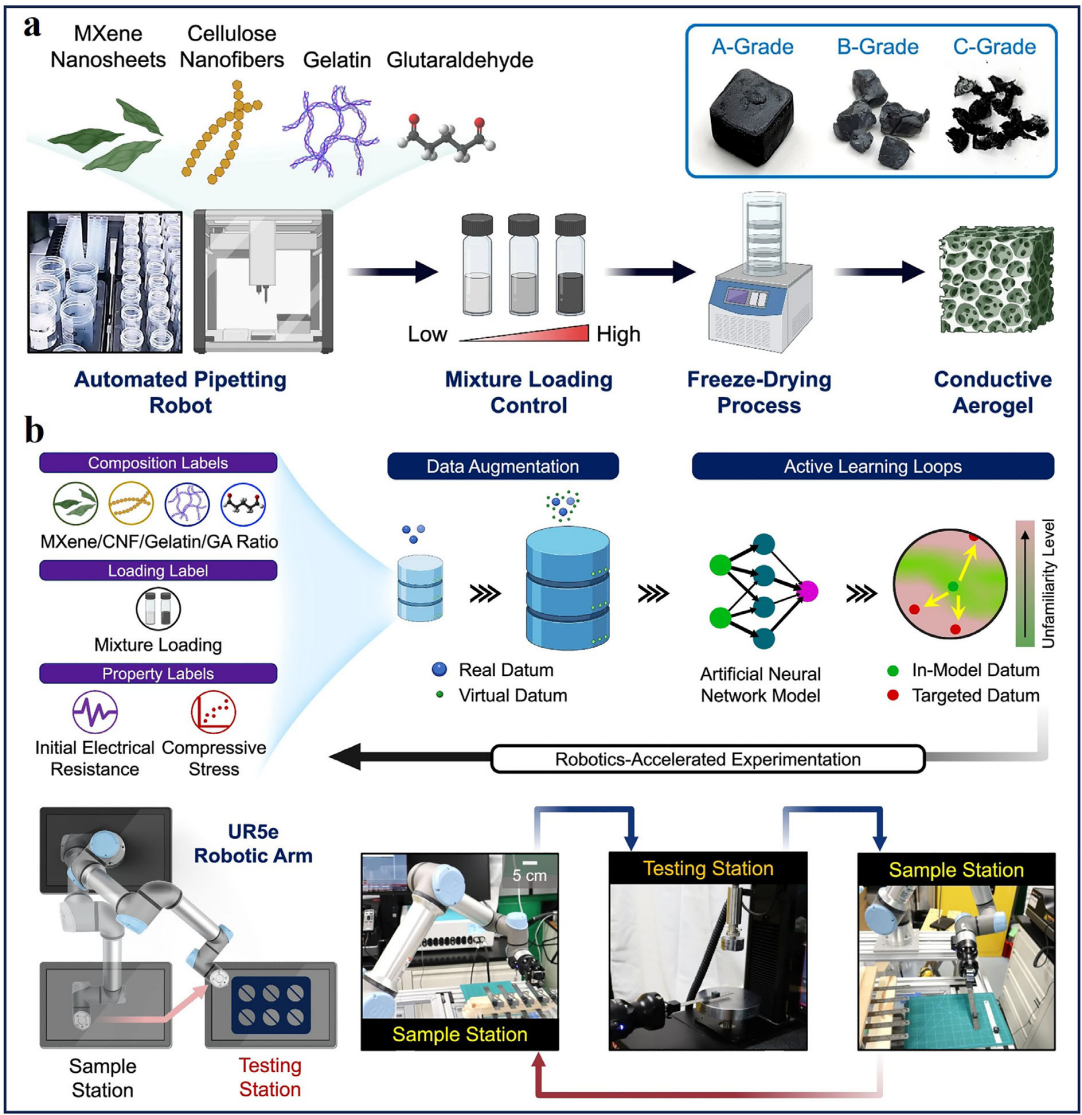
a) Schematic illustration, a high‐throughput synthesis pipeline by an automated pipetting robot (i.e., OT‐2 robot) for MXene mechanical and electrical properties modification. b) A robot‐human teaming workflow for MXene aerogel discovery, in which the data augmentation, active learning loops are integrated to refine prediction and guide subsequent experiments. The autonomous characterization platform utilizes a UR5e robotic arm, coupled with an Instron compression tester to enable automated sample handling and mechanical testing within the closed‐loop workflow [93]. Reproduced with permission [93]. Copyright 2024, Springer Nature.

The following robotic and high‐throughput study illustrate how automated execution, model‐guided selection, and experimental feedback can accelerate materials optimization. In a conductive MXene aerogel discovery pipeline [93], an OT‐2 robot pipetting robot was applied to rapidly generate 264 mixtures of MXene/CNF/gelatin formulations (CNF: cellulose nanofiber). The resulting aerogels were manually graded into intact monoliths (A‐grade), fragmented samples (B‐grade), and severely disrupted products (C‐grade), according to their structural integrity and monolithic character (Figure 11a). These graded data were subsequently used to train a support vector machine (SVM) classifier [126], which defined the decision boundaries separating formulations with different levels of synthetic viability. Therefore, the broad formulation space was firstly narrowed to a feasible design domain for subsequent active learning, which also ensured that the later property prediction was restricted to experimentally accessible and structurally meaningful samples [93].

The workflow was then extended into a collaborative robot‐assisted AI/ML framework. An AL algorithm was employed to identify informative formulations for subsequent experiments, while the OT‐2 robot continued to provide standardized formulation preparation and a UR5e collaborative robotic arm further enabled the automated sample handling during mechanical characterization. The newly acquired data, together with data augmentation, were thereby incorporated into the training set to build an ANN model within a high predictive accuracy. Once trained, the ANN model captured the mapping and established the synthesis‐property relationship of MXene aerogels, which then moved beyond forward prediction and enabled inverse design by identifying candidate formulations that satisfy predefined property targets (Figure 11b). Moreover, SHapley Additive exPlanations (SHAP) analysis, together with finite element simulation, provided a physics‐informed interpretive layer for the learned model by elucidating feature contributions and validating the effect of mixture loading on compressive behavior [127]. Overall, this study established a closed‐loop workflow that integrates collaborative robotics, feasibility‐informed learning, ANN‐based property prediction, and inverse design for the programmable MXene aerogel discovery.

Aside from robot‐human teaming workflow, the closed‐loop discovery frameworks have also been established by coupling automated laboratories with algorithmic decision‐making for the fully autonomous experiment selection, data execution and iterative optimization. In an early self‐driving system, the optimization variables are defined directly by experimentally operable synthesis parameters, allowing Bayesian Optimization (BO) to navigate a synthesis‐aware process space and return executable fabrication conditions [128]. This logic was further extended. The autonomous experimentation was combined with q‐Expected Hypervolume Improvement (qEHVI) so that new experiments are selected according to their expected contribution to the Pareto front, thereby turning the workflow into a function‐oriented multi‐objective search [129]. More recent inverse design strategies have become increasingly descriptor‐grounded [130], where chemically meaningful descriptors provide the basis for predictive modeling, BO recommends candidate materials toward predefined performance targets, and multitask Gaussian process regressors (MTGPR) capture and analyze the structure‐property relationships linking materials features to device performance [131]. This process can then be iteratively repeated to further expand the candidate space, coupled with high‐throughput platforms with more efficiency.

The methodological advance discloses the trend in moving beyond synthesis‐aware optimization of processing variables toward descriptor‐grounded inverse design, where the search is conducted in a chemically interpretable representation space that is more naturally aligned with function‐oriented materials discovery. Meanwhile, automated laboratories do not merely execute algorithm‐selected experiments, but continuously feedback the synthesis‐realistic data to replenish and refine the training space, making inverse design progressively more reliable and experimentally grounded [132]. Their direct transfer to LAMS‐derived MXenes remains constrained by high‐temperature molten salts, reactor compatibility, and multi‐step post‐treatment. A realistic path toward autonomous MXene discovery is therefore to first establish synthesis‐informed datasets and descriptor‐guided feedback workflows, while progressively developing molten salt compatible reactors and high‐throughput synthesis modules for future closed‐loop platforms.

## 5 Summary and Outlook

LAMS‐derived MXenes have rapidly expanded the accessible MXene design space by introducing broader compositional diversity, emerging structural architectures, and increasingly tunable surface chemistry through coupled surface engineering strategies. These characteristics make LAMS‐derived MXenes a particularly attractive platform for data‐driven materials discovery, as their synthesis‐dependent chemistry and structural complexity are difficult to systematically navigate using conventional trial‐and‐error approaches or brute‐force computation alone. Moving forward, an important direction will be to integrate the distinctive chemistry of LAMS‐derived MXenes with data‐driven methodologies capable of addressing synthesis‐dependent surface states, complex local configurations, and expanded functionalization spaces. Against this backdrop, several key perspectives for future development are highlighted.

### 5.1 Mapping Low‐Energy Eutectic LAMS Chemistries

Although LAMS offers a scalable, fluoride‐free, and functionally tailorable route to MXene synthesis, its relatively high operating temperature still imposes a substantial energy cost compared with solution‐based etching. A key future direction is therefore to establish broader mapping between the MAX‐Lewis acid redox chemistry matching and the etching thermodynamics, rather than relying on empirical pair selection alone. In particular, eutectic salt systems provide an important opportunity [133] because they can lower the effective reaction temperature while reshaping the thermodynamic and kinetic window of etching [9]. Coupling such chemistry‐informed design with ML screening may accelerate identification of favorable MAX/etchant/eutectic combinations and guide lower‐energy LAMS processes.

### 5.2 Bottom‐Up Precursor Design in LAMS‐Derived MXene

Another underexplored yet highly promising direction is to expand MXene design upstream to the MAX precursor stage, where rational matrix design [134] may already predetermine the composition and local chemical environment of the final LAMS‐derived MXene. Governed by molten‐salt thermodynamics and redox compatibility, such precursor‐to‐MXene inheritance extends MXene engineering beyond post‐synthetic regulation toward matrix‐level compositional programming. Data‐driven mapping of precursor composition, etching chemistry, and structural evolution may therefore accelerate discovery of precursor‐salt combinations that enable targeted MXene chemistries.

### 5.3 Data‐Driven Discovery of Intrinsic Metal States

Intrinsic metal species retained or generated during LAMS etching represent a distinct design dimension beyond the MXene host itself [135]. Their identity, coordination environment, and spatial distribution may directly regulate electronic structure, interfacial chemistry, and reactive microenvironments, creating opportunities for both direct functionality and in situ heterostructure formation. Because precursor chemistry, etching conditions, and coordination evolution are strongly coupled, ML‐assisted mapping of precursor–etching–structure relationships may help identify metal states and heterostructure motifs favorable for targeted functions. A practical near‐term goal is therefore to establish unified datasets linking residual metal descriptors with electrochemical performance under standardized conditions.

### 5.4 Large Language Models (LLMs) for Synthesis‐Informed MXene Discovery

For LAMS‐derived MXenes, LLMs may play a practical role in bridging scattered literature and model‐ready datasets [136]. Key information including etching chemistry, surface terminations, in situ coupling strategies, and post‐treatment histories remains largely embedded in unstructured reports rather than standardized databases [137]. LLM‐assisted extraction may improve data accessibility and descriptor construction [138], but reliable use still requires metadata standardization, source‐linked verification, and physics‐informed validation because inconsistent characterization and incomplete experimental reporting remain major limitations.

### 5.5 Interpretability for LAMS‐Derived MXene Screening

Electronic‐structure descriptors improve the physical relevance of MXene screening by capturing chemically meaningful surface and bonding information. The interpretability, however, lies in determining which of these descriptors actually control activity, how they are modulated by surface chemistry, and whether the resulting structure–function relations can be generalized into transferable design rules [138]. For LAMS‐derived MXenes, interpretability is more convincingly established when the whole synthesis process is explicitly resolved into a processing‐surface state‐electronic structure‐activity pathway. Such a framework can identify how precursor chemistry, Lewis‐acid redox matching, and termination evolution jointly determine the key descriptors that truly govern functionality.

### 5.6 High‐Throughput Platforms for LAMS‐Derived MXene

High‐throughput platforms are becoming increasingly important for data‐driven materials discovery. For LAMS‐derived MXenes, microfluidic and continuous‐flow systems may provide practical routes for downstream optimization of intercalation, washing, and post‐treatment variables [139, 140]. For upstream precursor engineering and high‐temperature validation, polymer pen lithography (PPL) [141] and especially scanning probe block copolymer lithography (SPBCL) [142] are particularly attractive because they combine ultrahigh‐throughput library fabrication [143] with local stoichiometric encoding [144]. Such approaches are conceptually compatible with thermally programmable LAMS chemistry and may ultimately enable chip‐scale screening of precursor‐salt‐temperature relationships under highly parallel conditions [145].

### 5.7 Inverse Design for LAMS‐Derived MXene Discovery

For LAMS‐derived MXenes, inverse design should move beyond nominal composition optimization, because target functionality is often governed by processing‐dependent surface states including surface terminations, coverage, local coordination, interlayer species and intrinsic metal modification. A more realistic framework is therefore to organize inverse design hierarchically [146]. In practice, the outer layer should begin with the target functionality and property window to narrow the search toward synthesis‐feasible precursor‐etchant‐processing candidates; the intermediate layer should map these chemistry and processing variables onto accessible structural/surface states; and the mechanistic layer should further connect these states to the predicted target properties and the key descriptors that govern functionality, with experimental validation and feedback iteratively updating each layer of the workflow [147]. From a data perspective, such a pipeline will require the coordinated use of high‐throughput computation for thermodynamics and descriptor generation [148], synthesis‐informed metadata extracted from literature and experimental records, structured feedback from high‐throughput experiments, and inverse models operating under synthesis constraints. Near‐term inverse design should begin with constrained targets, such as selecting precursor‐salt‐temperature conditions for stable halogen terminations or identifying post‐treatment windows that preserve interlayer structure while tuning residual metal states. These bounded tasks are more realistic than full inverse design across all LAMS‐derived compositions and heterointerfaces.

In summary, the chemical diversity of LAMS‐derived MXenes arises not only from composition, but also from synthesis‐dependent surface evolution involving termination chemistry, retained metal states, and heterointerface formation. This expanded chemical space creates both opportunities and challenges for MXene discovery, requiring descriptors that are physically meaningful and experimentally grounded. As synthesis, characterization, and data‐driven methodologies continue to mature, LAMS‐derived MXenes are expected to evolve from empirically optimized systems toward more predictive and chemistry‐informed materials design across diverse electrochemical applications.

## Conflicts of Interest

The authors declare no conflicts of interest.

## Data Availability

Data sharing not applicable to this article as no datasets were generated or analyzed during the current study.
